# MALT1 Auto-Proteolysis Is Essential for NF-κB-Dependent Gene Transcription in Activated Lymphocytes

**DOI:** 10.1371/journal.pone.0103774

**Published:** 2014-08-08

**Authors:** Mathijs Baens, Luca Bonsignore, Riet Somers, Charlotte Vanderheydt, Stephen D. Weeks, Jenny Gunnarsson, Ewa Nilsson, Robert G. Roth, Margot Thome, Peter Marynen

**Affiliations:** 1 Human Genome Laboratory, VIB Center for the Biology of Disease, Leuven, Belgium; 2 Human Genome Laboratory, Center for Human Genetics, KU Leuven, Leuven, Belgium; 3 Department of Biochemistry, University of Lausanne, Epalinges, Switzerland; 4 Laboratory for Biocrystallography, Department of Pharmaceutical and Pharmacological Sciences, KU Leuven, Leuven, Belgium; 5 Reagent and Assay Development, Discovery Sciences, Innovative Medicines, AstraZeneca R&D, Mölndal, Sweden; J. Heyrovsky Institute of Physical Chemistry, Czech Republic

## Abstract

Mucosa-associated lymphoid tissue 1 (MALT1) controls antigen receptor–mediated signalling to nuclear factor κB (NF-κB) through both its adaptor and protease function. Upon antigen stimulation, MALT1 forms a complex with BCL10 and CARMA1, which is essential for initial IκBα phosphorylation and NF-κB nuclear translocation. Parallel induction of MALT1 protease activity serves to inactivate negative regulators of NF-κB signalling, such as A20 and RELB. Here we demonstrate a key role for auto-proteolytic MALT1 cleavage in B- and T-cell receptor signalling. MALT1 cleavage occurred after Arginine 149, between the N-terminal death domain and the first immunoglobulin-like region, and did not affect its proteolytic activity. Jurkat T cells expressing an un-cleavable MALT1-R149A mutant showed unaltered initial IκBα phosphorylation and normal nuclear accumulation of NF-κB subunits. Nevertheless, MALT1 cleavage was required for optimal activation of NF-κB reporter genes and expression of the NF-κB targets IL-2 and CSF2. Transcriptome analysis confirmed that MALT1 cleavage after R149 was required to induce NF-κB transcriptional activity in Jurkat T cells. Collectively, these data demonstrate that auto-proteolytic MALT1 cleavage controls antigen receptor-induced expression of NF-κB target genes downstream of nuclear NF-κB accumulation.

## Introduction

The MALT1 gene was identified from a recurrent chromosomal translocation in mucosa–associated lymphoid tissue (MALT) lymphoma [1]. The t(11;18)(q21;q21) breakpoint generates an oncogenic API2-MALT1 fusion protein that constitutively activates NF-κB in cell lines [2], [3], in MALT lymphomas [4] and in transgenic mice [5]. Two other chromosomal translocations, t(1;14)(p22;q32) and t(14;18)(q32;q21), are also associated with MALT lymphoma and result in IgH enhancer-driven overexpression of BCL10 and MALT1 respectively [6]–[9]. Their oncogenic activity is linked to the involvement of the CARMA1-BCL10-MALT1 (CBM) complex in antigen receptor-mediated activation of the transcription factor NF-κB, which controls the expression of numerous anti-apoptotic and proliferation-promoting genes [10].

Genetic and biochemical studies have shown that MALT1 and its binding partner BCL10 act downstream of the scaffold protein CARMA1 (also known as CARD11) as key mediators of canonical NF-κB activation upon antigen receptor stimulation. Mice deficient for Bcl10 [11], Malt1 [12], [13] or Carma1 [14]–[17] display severely impaired T cell receptor (TCR) and B cell receptor (BCR) responses. Antigen triggering of T- and B-cells activates a cascade of tyrosine phosphorylation events that converge at the activation of Ser/Thr kinases such as PKCθ and PKCβ, respectively. Activated PKCθ/β (and most likely additional kinases) phosphorylate CARMA1, inducing a conformational change that exposes its coiled coil and CARD motifs [18], [19]. These events are thought to take place in lipid rafts, which are sphingolipid- and cholesterol-rich micro-domains in the cell membrane [20]. The phosphorylation-induced conformational change of CARMA1 allows the recruitment of additional CARMA1 molecules [18], BCL10 [19], [21], [22] and MALT1 [23] and most likely triggers the initiation of oligomeric active signaling complexes [24]. It is thought that the formation of CBM oligomers in turn induces the recruitment, oligomerization and activation of the E3-ubiquitin ligase activity of TRAF6, resulting in Lys63-linked poly-ubiquitination of MALT1 [25], BCL10 [26] as well as the ligase itself [27]. These poly-ubiquitin chains assist CARMA1-dependent recruitment of the IκB kinase (IKK) complex via the ubiquitin-binding domain of the IKKγ subunit [28], which then culminates in full IKK activation via poly-ubiquitination of IKKγ [29]. Activated IKK phosphorylates the NF-κB inhibitory protein IκB, which marks it for degradation by the proteasome, thereby releasing NF-κΒ complexes and allowing their nuclear translocation.

MALT1 controls T- and B-cell activation not only through its adaptor function but also via its proteolytic activity [30], [31]. TCR stimulation induces MALT1-mediated cleavage and inactivation of the NF-κB inhibitor A20, resulting in a stronger NF-κB response and increased IL-2 production [30]. Moreover, MALT1-dependent cleavage of RELB, an NF-κB family member that acts as a negative regulator of T-cell activation [32], promotes NF-κB activation in an IKK-independent manner [33]. To date four additional MALT1 substrates have been identified: BCL10, CYLD, MCPIP-1 (also known as Regnase-1) and NIK. Cleavage of MALT1's binding partner BCL10 does not control NF-κB activity but is thought to affect integrin-mediated T-cell adhesion [31]. Cleavage of CYLD, a de-ubiquitinating enzyme and known negative regulator of NF-κB signaling, was shown to be essential for TCR-induced JNK activation [34]. MCPIP-1 is an RNAse that destabilizes mRNAs of T cell effector genes; its cleavage by MALT1 leads to stabilization of TCR-induced gene transcripts [35]. Finally, cleavage of NIK by the API2-MALT1 fusion protein activates non-canonical NF-κB signaling, which contributes together with canonical NF-κB activation to MALT lymphomagenesis [36]. MALT1 protease activity is also essential for the survival of cells derived from the activated B-cell subtype of diffuse large B-cell lymphoma (ABC-DLBCL) [37], [38], which are addicted to constant MALT1-driven NF-κB signaling [39].

The MALT1 protein was originally referred to as a ‘paracaspase’ because it contains a caspase p20-like proteolytic domain preceded by a large pro-domain, consisting of a Death Domain (DD) and two immunoglobulin-like (Ig) domains [3]. As such, MALT1 structurally resembles initiator caspases. These have longer pro-domains and become catalytically active upon proximity-induced dimerization. Subsequent auto-proteolysis of the protease domain into p10 and p20 subunits is thought to stabilize activated caspase dimers [40]. Oligomerization of the CBM complex in the lipid raft environment after antigen-receptor triggering is thought to promote MALT1 proteolytic activity by induced proximity, similar to initiator caspases. However, the mechanisms required to stabilize the active form of MALT1 seem to be fundamentally different from initiator caspases. Indeed, catalytically active MALT1 dimers are stabilized by mono-ubiquitination of MALT1 on lysine 644 in its C-terminal region following the protease domain [41], [42]. Whether MALT1, like initiator caspases, is also a target of its own proteolytic activity, and if such an auto-proteolysis event could contribute to NF-κB signalling, is unknown. Here, we demonstrate that MALT1 is a substrate of its own protease activity and that MALT1 auto-proteolysis is an essential step in antigen receptor-induced NF-κB-dependent gene transcription.

## Results

### Targeting MALT1 to the plasma membrane in 293T cells induces its cleavage into 19 and 76 kDa fragments

**Figure 1:**
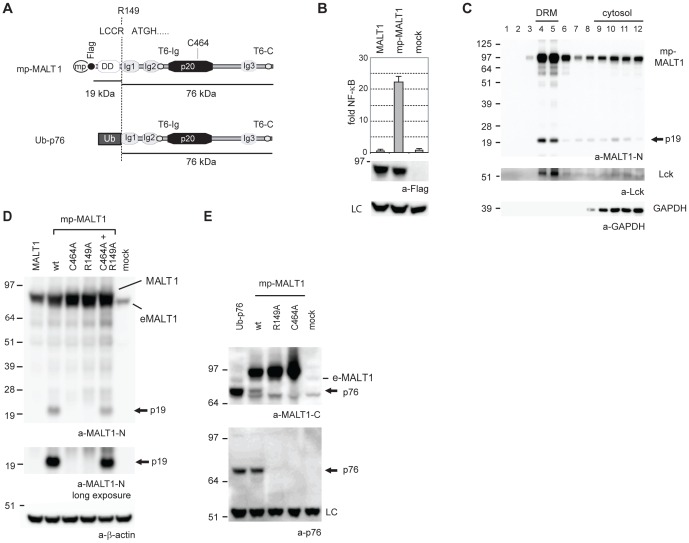
Targeting mp-MALT1 to DRMs induces its proteolysis at R149 in 293T cells. A) Features of mp-MALT1 (mp: myristoylation-palmitoylation sequence) and Ub-p76 (Ubiquitin-p76 fusion protein). R149: MALT1 cleavage site. Flag: Flag epitope, DD: Death Domain, Ig: immunoglobulin-like domain, p20: caspase p20-like domain, C464: MALT1 catalytic cysteine, T6-Ig and T6-C: TRAF6 binding site in second Ig domain and C-terminus, respectively. Ub: Ubiquitin. B) NF-κB-reporter assays of 293T cells transiently expressing wild-type MALT1, mp-MALT1 or empty vector (mock). NF-κB-dependent luciferase activity is shown as fold induction of vector-transfected cells and represents the mean +/- S.D. of at least three independent experiments (n = 3). Cell lysates were immunoblotted with a-Flag, a non-specific band was used as loading control (LC). C) Lysates of 293T cells transiently transfected with mp-MALT1 were subjected to sucrose density gradient centrifugation and aliquots of the serial fractions (1-12 from top to bottom) were immunoblotted with a-MALT1-N, a-Lck, a kinase residing in the Detergent Resistant Membrane (DRM) fractions, and a-GAPDH, a cytosolic marker. D-E) Immunoblot of lysates of 293T cells transiently expressing wild-type MALT1, mp-MALT1 and its mutants or Ubiquitin-p76 as specified with indicated antibodies. eMALT1: endogenous MALT1. β-actin (D) and LC: non-specific band (E) are loading controls. Arrows (panel C, D, E)) indicate the N-terminal p19 or the C-terminal p76 cleavage fragment respectively. All molecular mass standards are in kDa.

T or B cell receptor stimulation induces CARMA1-mediated recruitment of BCL10 and MALT1 to the lipid raft membrane fractions, which is essential for NF-κB activation [14], [22], [43]. To test whether lipid raft targeting promotes MALT1 activation, we generated a fusion of MALT1 to the N-terminal myristoylation-palmitoylation signal sequence of Lck (mp-MALT1, Figure 1A), which can target proteins into glycosphingolipid-enriched membranes [44]. When ectopically expressed in 293T cells, this construct was highly active, while MALT1 alone was unable to activate an NF-κB reporter ([45] and Figure 1B). We demonstrated previously through subcellular fractionation via sucrose density gradient centrifugation that ectopic MALT1 in 293T cells is merely cytosolic [5]. Subcellular fractionation of mp-MALT1 showed that it, as expected, also resides in the detergent resistant membrane (DRM) fractions, the latter marked by the presence of the kinase Lck (Figure 1C, lane 4 and 5). With the MALT1 antibody used (MALT1-N), which recognizes the N-terminus of MALT1, we further detected a 19 kDa fragment (p19) in the DRM fractions, (Figure 1C, lane 4–5). This N-terminal p19 fragment was also detectable in lysates of 293T cells expressing mp-MALT1, though not in lysates of cells expressing wild-type MALT1 (Figure 1D, lane 1–2). Expression of a catalytically inactive form of MALT1 (mp-MALT1-C464A) failed to generate the p19 fragment (Figure 1D, lane 3), suggesting that MALT1 protease activity is required for the generation of this N-terminal fragment.

MALT1 protease has specificity for an arginine (R) residue in the substrate P1 position [30], [31], [46]. Mutating candidate cleavage sites in mp-MALT1 showed that an R149A mutant was resistant to cleavage, similar to the C464A mutant (Figure 1D, lane 3-4), while normal cleavage occurred for an R191A mutant (Figure S1 A, lane 4). Like mp-MALT1, the R149A mutant cleaved known MALT1 substrates A20 and CYLD with comparable efficiency in 293T cells and had normal enzymatic activity in a cellular YFP-LVSR-CFP reporter cleavage assay (Figure S1 B and C). Co-expression of the R149A mutant of mp-MALT1 with the C464A mutant restored p19 formation again (Figure 1D, lane 5). This indicates that the catalytic activity of the R149A mutant can mediate - directly or indirectly - the cleavage of the inactive C464A mutant of mp-MALT1 after R149.

Western blotting with an antibody directed against the MALT1 C-terminus identified a 76 kDa fragment (p76) for mp-MALT1, which was absent for the R149A and C464A mutants (Figure 1E, top). This finding is consistent with cleavage of mp-MALT1 (95 kDa) into two fragments of 19 and 76 kDa, respectively. The p76 fragment generated from mp-MALT1 was also detected with an antibody raised against the p76 neo-epitope (Figure 1E, bottom, lane 2). A ubiquitin-p76 fusion protein, which is efficiently processed by ubiquitin-specific proteases in the cell into free ubiquitin and the p76 neo-epitope fragment, served as a positive control for p76 generation (Figure 1A and 1E, lane 1). Taken together, these data suggest that targeting of mp-MALT1 to DRMs induces its protease activity, and that MALT1 activity is required for its cleavage at R149 in the pro-domain.

### BCL10 induces cleavage of MALT1 at R149 in 293T cells

**Figure 2:**
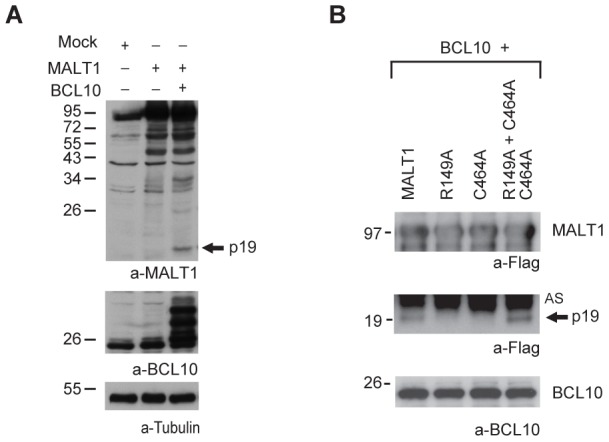
BCL10 mediates cleavage of MALT1 at R149 in 293T cells. A) Immunoblot of lysates of 293T cells transiently expressing MALT1 alone or in combination with BCL10 with antibodies against MALT1 [31], BCL10 and tubulin. B) Immunoblot of streptavidin pull-downs (bio-IPs) of Avi-tagged MALT1 and its mutants co-expressed with BCL10 in 293T cells as specified. AS: a-specific band. Arrows indicate the N-terminal p19 cleavage fragment. All molecular mass standards are in kDa.

Lucas *et al.* [2] reported that co-expression of BCL10 resulted in strong MALT1 oligomerization and activation of an NF-κB reporter in 293T cells [2]. We and others had demonstrated that co-expression of BCL10 with MALT1 in 293T cells triggers MALT1 protease activity and cleavage of its substrates A20 and BCL10 [30], [31]. When co-expressing BCL10 and MALT1 in 293T cells, we again observed the appearance of the p19 N-terminal cleavage fragment of MALT1 (Figure 2A, lane 3), in addition to a previously described hyper-phosphorylation of BCL10 [30], [31]. Processing did not occur when BCL10 was co-expressed with the C464A or the R149A mutant of MALT1 separately (Figure 2B). However, formation of the p19 fragment was restored when the R149A and C464A mutants were co-expressed together with BCL10, indicating that in this experimental setting, BCL10 triggers the protease activity of the MALT1-R149A mutant that induces – directly or indirectly - the cleavage of the MALT1-C464A mutant at R149 (Figure 2B).

### MALT1 undergoes auto-proteolysis *in vitro*

**Figure 3:**
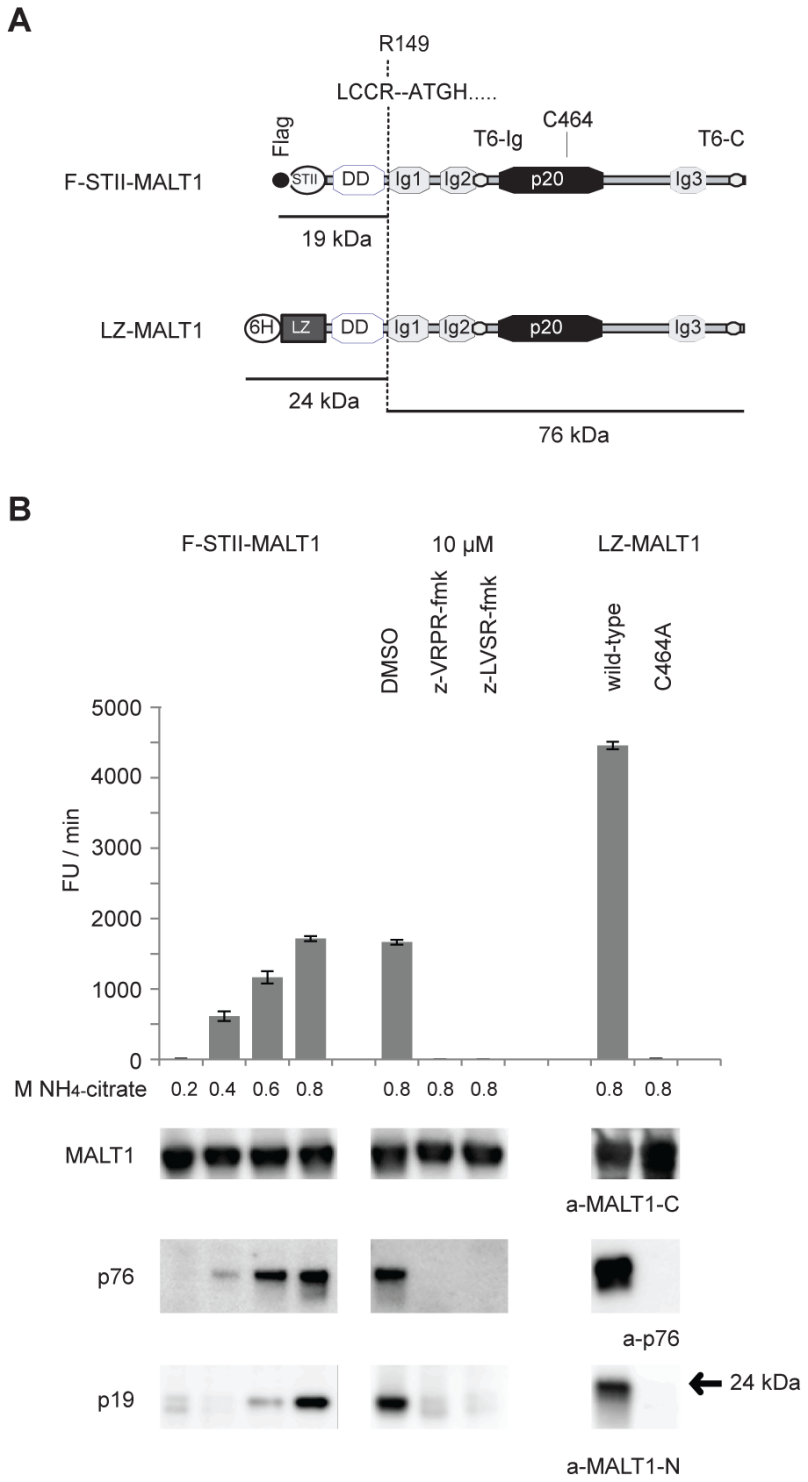
MALT1 undergoes auto-proteolysis *in vitro*. A) Features of F-STII-MALT1 and LZ-MALT1. F: Flag epitope, STII: StrepII-tag, 6H: 6-Histidine tag, LZ: Leuzine zipper. B) Top: *In vitro* cleavage of the fluorogenic tetratpeptide substrate Ac-LVSR-AMC (50 µM) by F-STII-MALT1 in increasing concentrations of the cosmotropic salt NH4-citrate (0.2, 0.4, 0.6, and 0.8 M M), by F-STII-MALT1 in 0.8M NH_4_-citrate buffer in the presence of the MALT1 protease inhibitors z-VRPR-fmk (10 µM) and z-LVSR-fmk (10 µM) and by LZ-MALT1 or LZ-MALT1-C464A in 0.8 M NH M NH_4_-citrate buffer. The barchart shows cleavage activity as Fluorescence Units (FU) increase/min. Results are expressed as means ± SD (n = 3). Bottom: enzymatic reactions were analysed by immunoblotting with a-MALT1-C, a-p76 neo-epitope and a-MALT1-N.

Thus far MALT1 cleavage was observed in cellular assays, which do not discriminate between a direct, auto-proteolytic event and an indirect cleavage event, which could be mediated by a protease that is activated by MALT1-mediated processing. To test the possibility of MALT1 auto-proteolysis we therefore performed *in vitro* cleavage assays. The recombinant MALT1 we used represents the full length MALT1 with the Flag-epitope and the StrepII-tag at its N terminus (F-STII-MALT1, Figure 3A). This construct was purified from stably transduced HKB11 cells via Strep-Tactin affinity chromatography. The *in vitro* proteolytic activity of F-STII–MALT1 was assessed by incubation with the fluorogenic tetrapeptide substrate Ac-LVSR-AMC in paracaspase assay buffer [33]. Increasing concentrations of the cosmotropic salt NH_4_-citrate gradually increased MALT1 cleavage activity and release of free AMC, which could be completely blocked by the MALT1 tetrapeptide inhibitors z-VRPR-fmk and z-LVSR-fmk (Figure 3B, top). Immunoblots of the *in vitro* reactions further demonstrated generation of the p76 and p19 cleavage fragments of MALT1 with an efficiency that mimicked the pattern of MALT1 protease activity observed in the LVSR-AMC protease assay (Figure 3B and S3, bottom).

To further assess MALT1 auto-proteolysis, we generated recombinant forms of MALT1 (aa 2-824) fused to a leucine zipper dimerization motif (LZ-MALT1 and LZ-MALT-C464A) (Figure 3A), which promotes dimerization-dependent MALT1activation [47]. Processing of Ac-LVSR-AMC by LZ-MALT1 in the presence of 0.8 M NH_4_-citrate was 3 fold higher than for F-STII-MALT1, whereas a corresponding LZ-MALT1-C464A construct was inactive (Figure 3B, top). Immunoblots of the *in vitro* cleavage reactions again showed the generation of the expected N-terminal fragment of 24 kDa (matching the p19 fragment) and the C-terminal p76 fragment for LZ-MALT1 though not for its C464A mutant (Figure 3B, bottom). Collectively these data indicate that MALT1 is able to cleave itself at R149.

### The MALT1 p76 cleavage fragment activates NF-κB signalling

**Figure 4:**
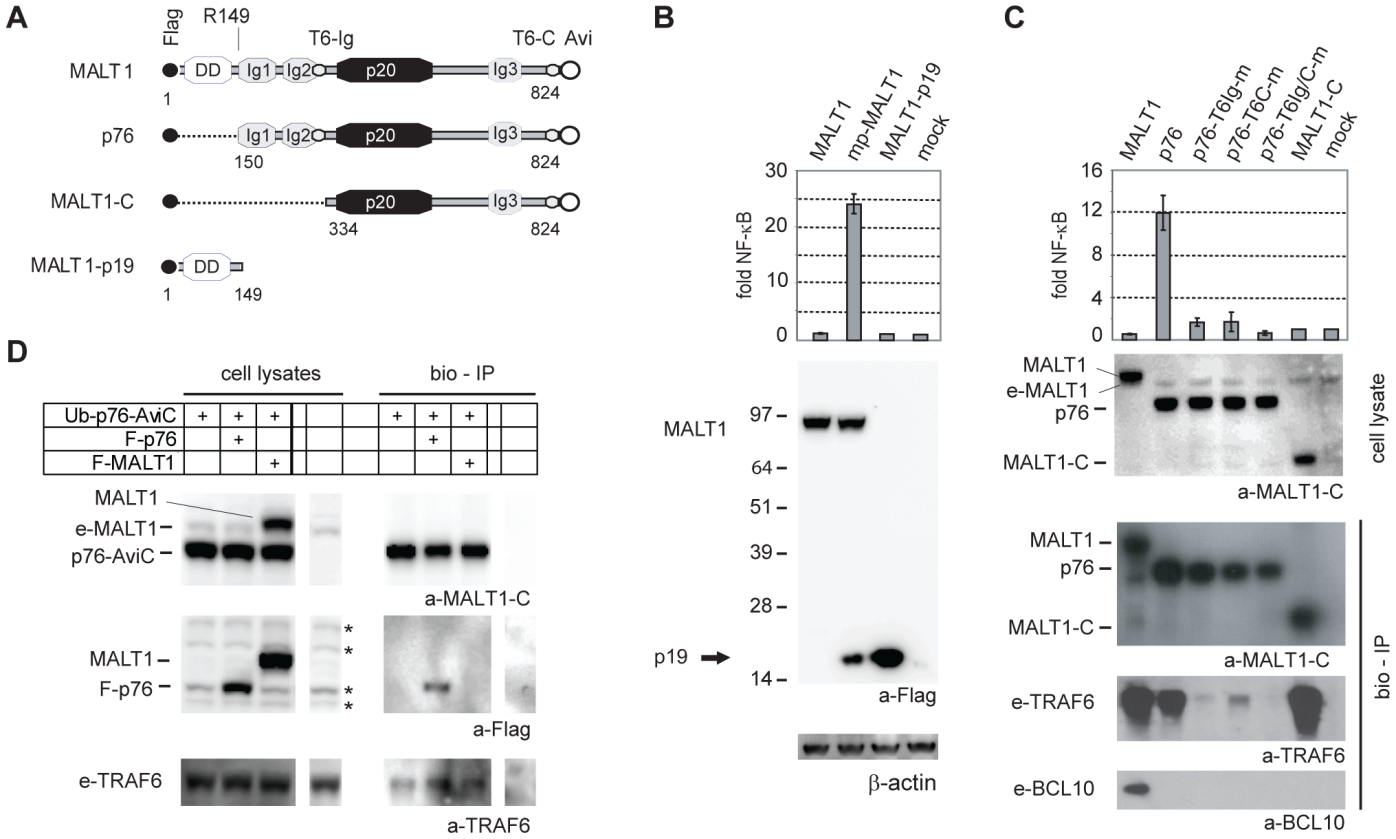
MALT1 p76 activates NF-κB signalling in 293T cells. A) Features of Flag-tagged MALT1, p76, MALT1-C and MALT1-p19. Numbers indicate the start and stop AA position for p76, MALT1-C and MALT1-p19 relative to the MALT1 protein sequence (Refseq NP_006776.1, 824 AA). B-C) NF-κB-reporter assays of 293T cells transiently expressing MALT1, mp-MALT1, MALT1-p19, p76 and mutants or MALT1-C. NF-κB-dependent luciferase activity is shown as fold induction of vector-transfected cells and represents the mean +/- S.D. (n = 3). Immunoblot of cell lysates with a-Flag – a-β-actin shows equal expression/loading of the different MALT1 constructs (B) and a-MALT1-C (C) shows equal expression of the different MALT1 constructs. Bottom (C): streptavidin pull-down (bio-IP) of MALT1, p76 or MALT1-C, transiently expressed in 293T cells, and immunoblotted with a-MALT1C, a-TRAF6 and a-BCL10 antibodies. e-MALT1, e-TRAF6, e-BCL10: endogenous MALT1, TRAF6 and BCL10. D) immunoblot of bio-IPs of Avi-tagged Ub-p76 expressed together with Flag-p76 or Flag MALT1 with a-MALT1-C, a-Flag and a-TRAF6. * indicate non-specific bands.

To investigate whether auto-processing of MALT1 has a role in NF-κB signalling, we first tested the capacity of the p19 and p76 fragments of MALT1 to promote NF-κB activation. Like full length MALT1 alone, expression of the p19 fragment (MALT1-p19, Figure 4A) did not activate a NF-κB reporter in 293T cells (Figure 4B). In contrast, the p76 fragment potently activated the NF-κB reporter despite the fact that it has lost the ability to bind BCL10 (Figure 4C). This suggests that removal of the N-terminal part of MALT1 might promote its capacity to activate NF-κB in a BCL10-independent manner. NF-κB activation by MALT1 involves TRAF6 binding via two distinct binding sites [27], [45] located within the Ig2 domain (T6-Ig) and at the MALT1 C-terminus (T6-C), respectively, and both are present in the p76 cleavage fragment (Figure 4A). Mutation of either one of the two TRAF6 binding sites, E313A/E316A (T6Ig-m) or E806A (T6C-m), strongly impaired the potential of p76 to activate NF-κB signalling in 293T cells (Figure 4C). Steptavidin pull-down experiments with Avi-tagged p76 constructs (bio-IP) confirmed that each of these individual mutations severely weakened the p76/TRAF6 interaction, while a complete inhibition of TRAF6 binding required mutation of both sites (T6Ig/C-m) (Figure 4C, bottom). Thus, p76-mediated NF-κB activation was clearly TRAF6-dependent. A shorter MALT1-C construct comprising only AA 334 to 824, which retained efficient TRAF6 binding via the T6C binding site, was unable to activate NF-κB signalling, suggesting an additional requirement for the intact Ig1 and Ig2 domains (Figure 4, A and C). Collectively, these data suggest that the MALT1 p76 fragment promotes NF-κB activation in a TRAF6-dependent but BCL10-independent manner.

Activation of NF-κB signalling downstream of MALT1 involves the E3 ubiquitin ligase activity of TRAF6, which is activated upon TRAF6 oligomerization [27]. Analysis of the structure of the tandem Ig1–Ig2 domains of MALT1 by crystallography and size exclusion chromatography has suggested their potential to form tetramers [48]. However, no oligomerization has been observed when full-length MALT1 proteins were expressed by themselves [2], suggesting that the N-terminal DD domain of MALT1 might prevent oligomerization. Bio-IP experiments with Avi-tagged p76 indeed showed an interaction with Flag-tagged p76, but not with full-length MALT1 (Figure 4D). Auto-proteolytic removal of the DD domain of MALT1 might thus facilitate oligomerization of p76 and the associated TRAF6 molecules, thereby inducing the E3 ubiquitin ligase activity of the latter required for downstream signalling.

### MALT1 is cleaved in stimulated B cells and in ABC-DLBCL cells

**Figure 5:**
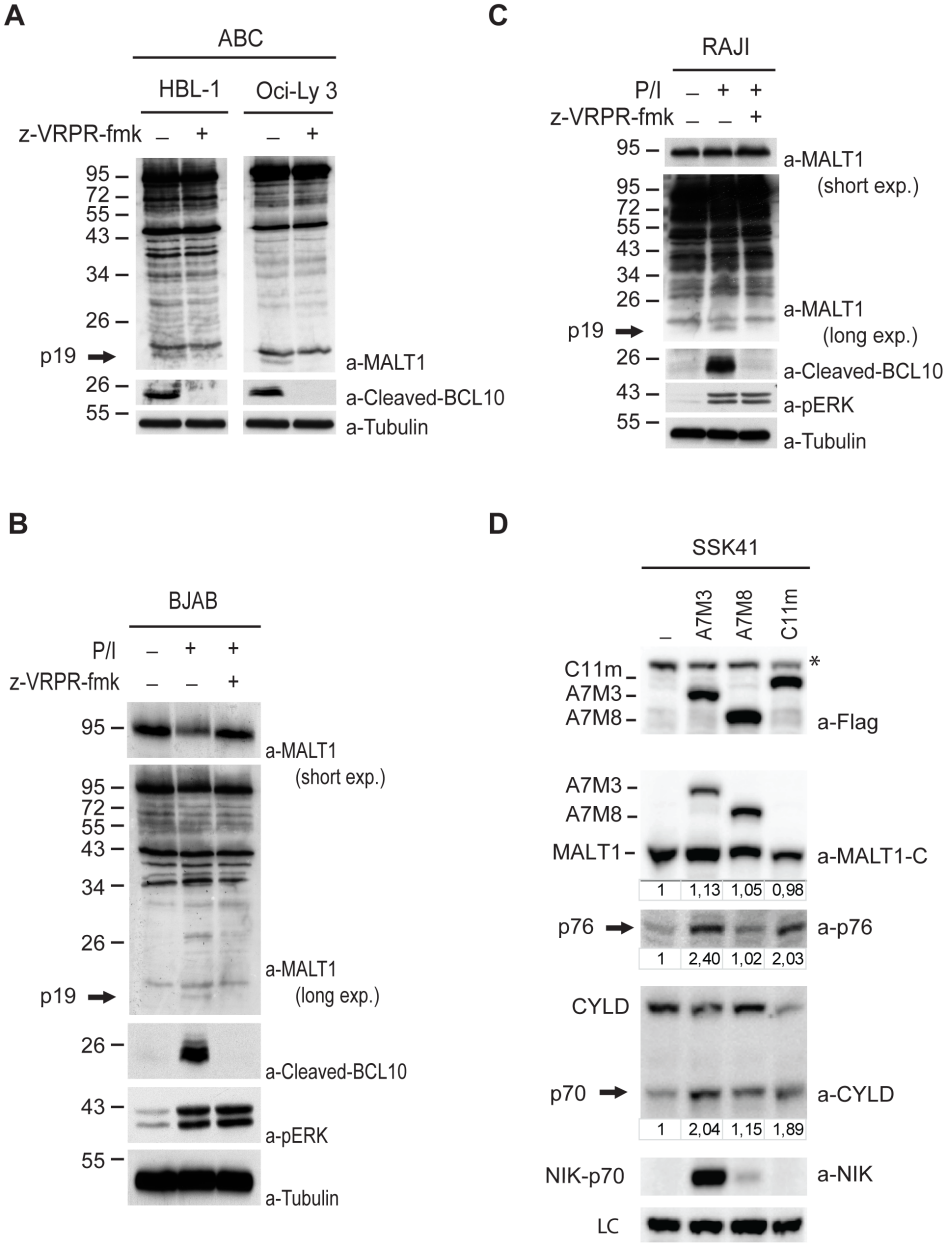
Figure 5. MALT1 auto-proteolysis in activated B cells. A) ABC-DLBCL cell lines HBL-1 and OCI-Ly3 were treated with 50 µM z-VRPR-fmk (36 hrs hrs) and lysates were analysed for the presence of MALT1 and BCL10 cleavage fragments with a-MALT1, a-Cleaved BCL10 and a-Tubulin (loading control). B-C) The GCB-DLBCL cell lines BJAB and Raji were left untreated or stimulated with PMA/ionomycin (30 min min) with or without pre-treatment with 50 µM z-VRPR-fmk (30 min min). Lysates were analysed for the presence of MALT1 and BCL10 cleavage fragments, for p-ERK (activation control) and tubulin (loading control). D) Immunoblot of lysates of SSK41 cells and SSK41 cells with ectopic expression of the API2-MALT1 fusion variants A7M3 and A7M8, or the L232LI mutant of Card11 (C11m) respectively, with antibodies against the MALT1 C-terminus, the p76 neo-epitope, the CYLD C-terminus, the NIK C-terminus and Flag (ectopic A7M3, A7M8 and C11m). Numbers below blots depict band intensities of MALT1, p76 and the CYLD p70 fragment relative to lane 1. *  =  non-specific band. LC: loading control, a non-specific band obtained with the p76 neo-epitope antibody was used.

Next we investigated the occurrence of MALT1 proteolysis in cell lines derived from DLBCL. The activated B-cell (ABC)-subtype of DLBCL is addicted to NF-κB signalling [49] and has constant MALT1 protease activity [37], [38]. Consequently, cell lines derived from such lymphomas, such as HBL-1 and OCI-Ly3, show a constitutive presence of cleaved BCL10 that can be detected with an antibody specifically recognizing the processed form of this protein [38] (Figure 5A). Likewise, the MALT1 p19 fragment was detected in lysates of these cells, and both MALT1 and BCL10 cleavage could be prevented by treating the cells with the MALT1 protease inhibitor z-VRPR-fmk (Figure 5A). In contrast, no MALT1 processing was detectable in the B cell lymphoma cell line BJAB, which is derived from the germinal center B-cell (GCB) subtype of DLBCL and has no steady MALT1 protease activity (Figure 5B). MALT1 processing was also undetectable in the EBV-transformed B-cell line Raji (Figure 5C). However, stimulation of these cells with PMA and ionomycin (P/I) induced MALT1 protease activity and the appearance of cleaved BCL10 and the MALT1 p19 fragment, which again could be blocked by addition of z-VRPR-fmk (Figure 5, B and C).

The marginal zone B cell lymphoma cell line SSK41 has an amplification of the MALT1 locus [8] that drives its overexpression and has constant MALT1 protease activity. As a consequence, SSK41 cells constantly cleave MALT1 substrates A20 [30] and CYLD ([34] and Figure 5D, lane 1). Western blot analysis further showed persistent MALT1 proteolysis in SSK41 cells generating the p76 (Figure 5D) and the p19 fragments (Figure S2A). MALT1 protease activity could be further increased in SSK41 cells via stable expression of an oncogenic Card11-L232LI (C11m) mutant [50], yielding increased processing of MALT1 and CYLD (Figure 5D, lane 4). Similarly, stable expression of the API2-MALT1 fusion variants A7M3 and A7M8 (which result from fusion of exon 7 of API2 with exon 3 or 8 of MALT1, respectively) not only induced cleavage of the API2-MALT1-specific substrate NIK [36], but it also increased the levels of cleaved CYLD (Figure 5D, lane 2 and 3). Both NIK and CYLD were more efficiently cleaved by the A7M3 variant than by A7M8, suggesting that the Ig1-Ig2 domains somehow enhance the protease activity of A7M3. We further noticed increased levels of MALT1 p76 in SSK41-A7M3 cells in contrast to A7M8 expressing cells (Figure 5D, lane 2 and 3). The A7M3 fusion contains the R149 cleavage site of MALT1, suggesting that A7M3 proteolysis might contribute to the increased p76 levels (Figure S2B). Western blot analysis of lysates of 293T cells expressing A7M3 indeed showed both the C-terminal p76 fragment and the anticipated N-terminal fragment of 54 kDa, which were absent for an A7M3-R149A mutant (Figure S2C). Again, the R149A mutation did not affect the protease activity of A7M3 as cleavage of A20 and CYLD were unaffected (Figure S2C). Altogether, these data demonstrate that MALT1 undergoes auto-processing in lymphoma cells as a consequence of either constitutive upstream signals promoting MALT1 activation (such as in ABC DLBCL expressing oncogenic CARMA1) or a genetic fusion of MALT1 to the apoptosis inhibitor API2, which results in the formation of a hyperactive oncogenic API2-MALT1 fusion protein.

### MALT1 auto-proteolysis is required for optimal IL-2 production in Jurkat T cells

**Figure 6:**
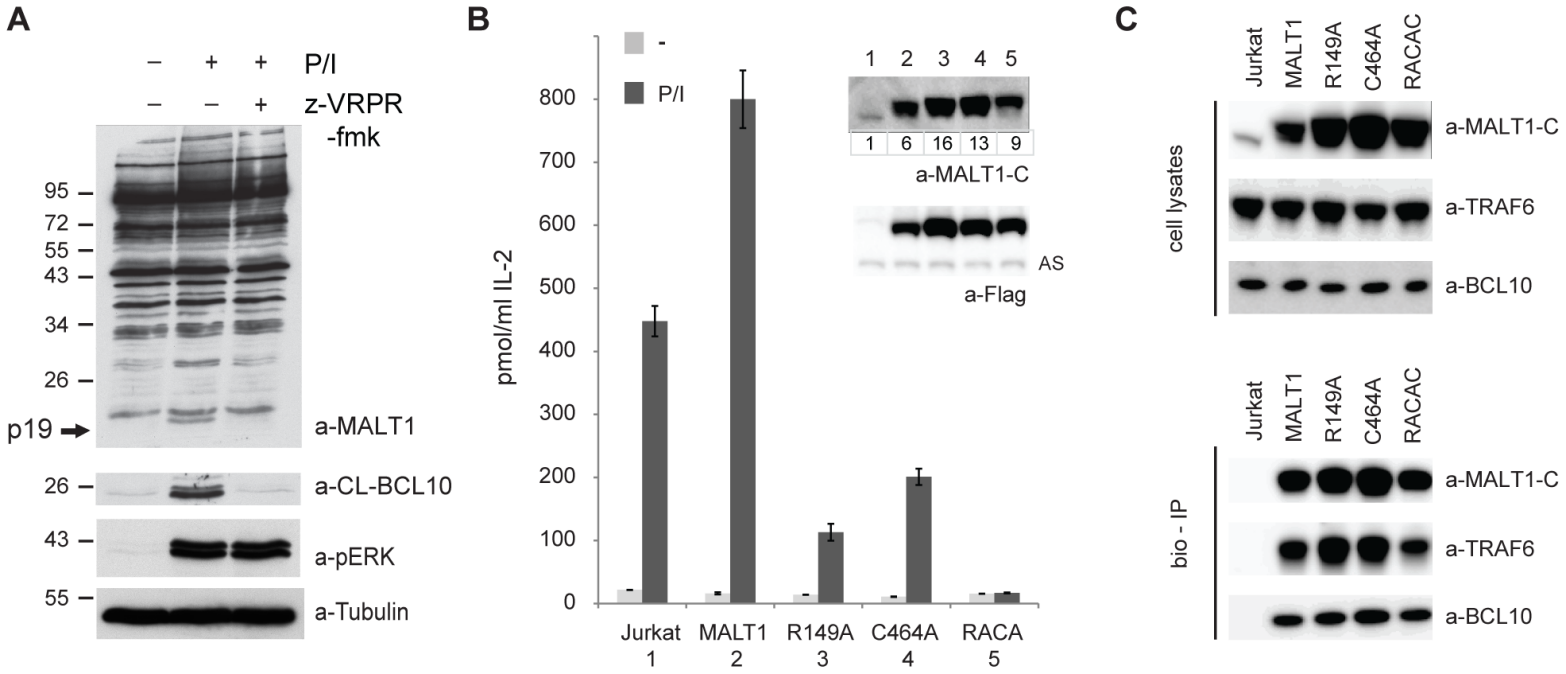
MALT1 auto-proteolysis is required for IL-2 production by Jurkat T cells. A) Jurkat T cells were left untreated or stimulated with P/I for 30 min, with or without pre min, with or without pre-treatment with 50 µM z-VRPR-fmk for 30 min. Lysates were analysed for the presence of the cleavage fragments for BCL10 and MALT1 p19, for p min. Lysates were analysed for the presence of the cleavage fragments for BCL10 and MALT1 p19, for p-ERK (activation control) and tubulin (loading control). B) IL-2 production (ELISA) of Jurkat T cells stably expressing MALT1, MALT1-R149A, MALT1-C464A or MALT1-RACA, either untreated (-) or stimulated for 18 hrs with PMA hrs with PMA/ionomycin (P/I). Data shown as mean +/- S.D. (n = 3). Inset: Immunoblot with a-MALT1-C and a-Flag showing expression of ectopic MALT1 and mutants relative to endogenous MALT1 (lane 1). Numbers indicate fold overexpression relative to endogenous MALT1. AS: a-specific band obtained with a-Flag that serves as loading control. C) Immunoblot of cell lysates (top) and bio-IPs (bottom) from Jurkat T cells and Jurkat T cells with stable expression of Avi-tagged MALT1 or MALT1 mutants R149A, C464A and RACA with indicated antibodies.

Next, we investigated whether MALT1 auto-processing affects T-cell activation. Stimulation of Jurkat T cells induced MALT1 protease activity, as demonstrated by the appearance of cleaved BCL10 after 30 minutes of P/I stimulation (Figure 6A). Simultaneously, the MALT1 p19 fragment was detected in lysates of these cells, and both cleavage events could be prevented by treatment of the cells with the MALT1 protease inhibitor z-VRPR-fmk (Figure 6A). To assess the relevance of MALT1 cleavage in T-cell activation, we generated Jurkat T cells that overexpress wild-type MALT1, the cleavage insensitive R149A mutant, the catalytically inactive C464A mutant or the double R149A/C464A mutant (RACA). Stimulation of these cells with P/I showed that none of the mutants affected the levels of inducible phosphorylation of IκBα or JNK or the nuclear accumulation of NF-κB subunits (Figure S4), events that are known to depend on the scaffold function of MALT1. Moreover, and in contrast to the C464A and RACA mutants, the R149A mutant did not affect cleavage of the MALT1 substrates A20, CYLD or RELB (Figure S4). Similar observations were made in the C464A and R149A cells in which endogenous MALT1 was inactivated using TALENs that target exon 2 of *MALT1* (Figure S5 and S6).

Next, we tested the role of MALT1 auto-processing in the expression of the NF-κB target gene IL-2. Compared to cells expressing exogenous wild-type MALT1, the stimulation-induced IL-2 secretion of the MALT1-R149A and -C464A mutant-expressing cells was reduced by 85 and 75%, respectively, indicating an important role for MALT1 cleavage at R149 in the IL-2 production of activated Jurkat T cells (Figure 6B). Nevertheless, all constructs showed comparable binding to BCL10 and TRAF6 (Figure 6C). Residual IL-2 production by Jurkat T cells expressing MALT1-C464A or R149A might have resulted from endogenous MALT1 that, upon stimulation, is able to cleave the C464A mutant or is cleaved by the R149A mutant, thereby generating the p76 fragment. Consistent with this hypothesis, stable expression of the MALT1-RACA double mutant, which excludes both possibilities, led to an almost complete inhibition of IL-2 production by stimulated Jurkat T cells (Figure 6B), suggesting that MALT1 auto-proteolysis might occur mainly in trans. Collectively, these data point towards a unique role for MALT1 auto-proteolysis and the resulting p76 fragment of MALT1 in T-cell activation.

### MALT1 auto-proteolysis is required to induce the transcription of NF-κB target genes

**Figure 7:**
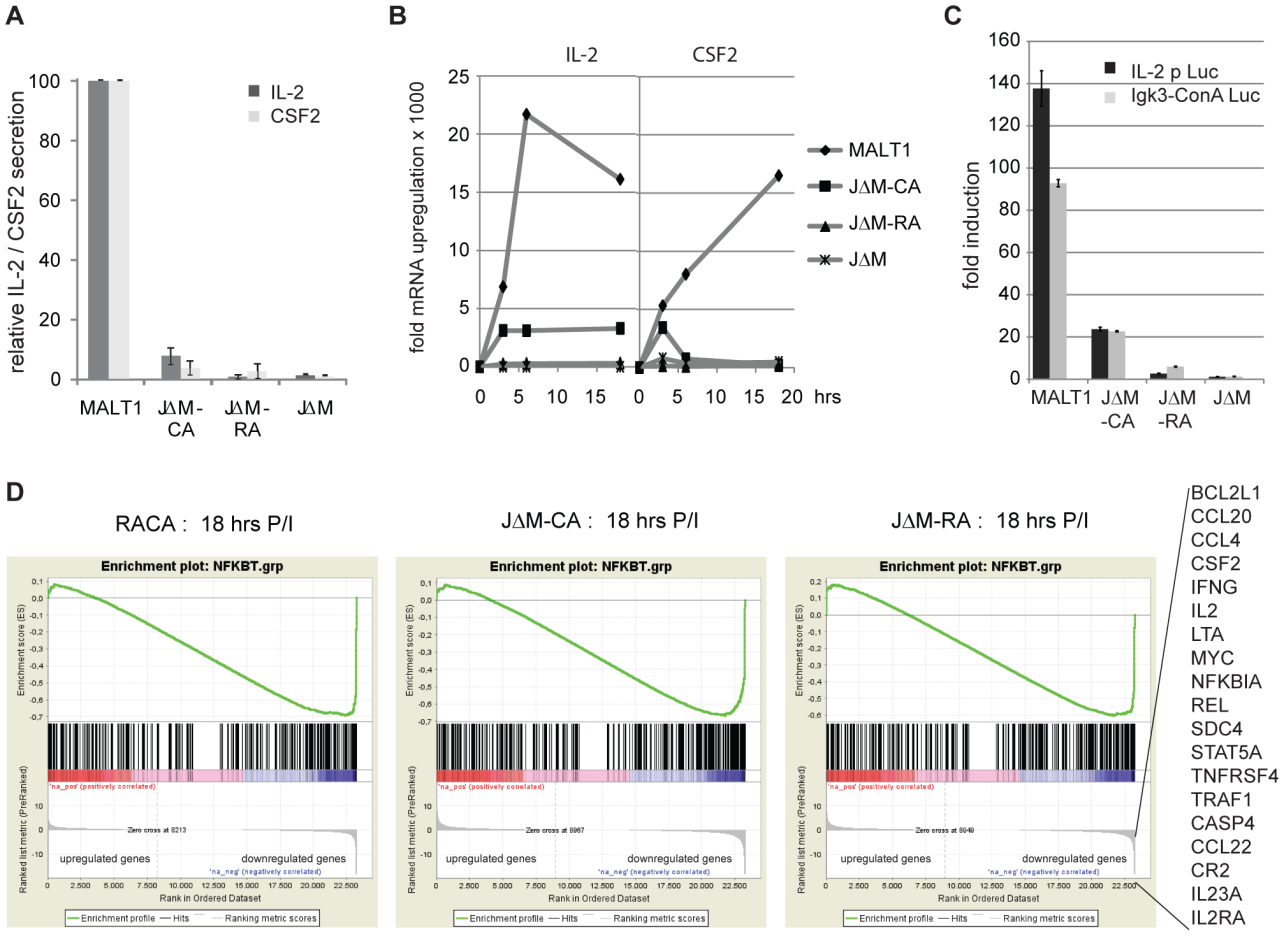
MALT1 auto-proteolysis is required for NF-κB transcriptional activity in Jurkat T cells. A) Relative IL-2 and CSF2 production of indicated Jurkat T cell lines, stimulated for 18 hrs with PMA hrs with PMA/ionomycin, measured via ELISA. Data shown as mean +/- S.D. (n = 3). B) Jurkat T cells expressing MALT1 and the JΔM-CA, JΔM-RA, and JΔM cells were stimulated for the indicated times with P/I and IL-2 and CSF2 transcript levels were determined via qRT-PCR. C) Jurkat T cells with ectopic expression of MALT1 and JΔM-CA, JΔM-RA and JΔM were electroporated (Amaxa, Nucleofection) with Luciferase reporter constructs driven by the IL-2 promoter or the Igκ3-ConA promoter, and 24 hrs later stimulated with P hrs later stimulated with P/I for 18 hrs before Luciferase activity was measured. Data shown as mean +/- S.D. (n = 3). D) GSEA showing a significant enrichment of NF-κB-target genes (FDR q<0,001) in the pre-ranked down-regulated genes from JΔM-CA, JΔM-RA and RACA cells stimulated for 18 hrs with P hrs with P/I. Gene list depicted at the right side are NF-κB target genes down-regulated in JΔM-CA, JΔM-RA and RACA cells after 3 and 18 hrs stimulation with P hrs stimulation with P/I.

So far, our data suggested that MALT1 auto-proteolysis affects neither IKK activation nor the nuclear translocation of NF-κB, despite a profound defect on the expression of the NF-κB target gene IL-2. Bi-allelic inactivation of endogenous MALT1 in R149A cells (JΔM-RA) further reduced IL-2 as well as CSF2 secretion to the basal levels observed in Jurkat T cells with bi-allelic MALT1 inactivation (JΔM) (Figure 7A). To assess whether these defects in IL-2 and CSF2 secretion were due to defects in transcription, we performed qRT-PCR analysis. A stimulus-dependent up-regulation of IL-2 and CSF2 mRNA levels was observed in the MALT1 expressing Jurkat T cells, while all Jurkat mutants were seriously hampered in their mRNA up-regulation (Figure 7B). Next we performed luciferase reporter assays with the different Jurkat clones. Cells expressing wild-type MALT1 showed a stimulus-dependent increase of gene reporter constructs containing the IL-2 promotor (IL-2p-Luc) or three NF-κB sites from the promoter of the kappa light chain of immunoglobulin (Igκ3-ConALuc), while this response was strongly impaired in JΔM-CA or JΔM-RA cells (Figure 7C). Together, these findings clearly support a role for MALT1 auto-proteolysis in regulating NF-κB transcriptional activity. To explore this further, we performed RNA sequencing for stimulated Jurkat MALT1 cells and the mutant RACA, JΔM-CA and JΔM-RA cells. Compared to the Jurkat MALT1 cells, the three mutant cell lines showed between 79 and 278 differentially expressed genes (DEGs) with a greater than two-fold change and a false discovery rate (FDR) q<0.001 after 3 or 18 hrs of stimulation with P/I (Table S2). qRT-PCR analysis performed for a selection of top ranked genes confirmed the effects observed by RNA sequencing (Table S3). Gene Set Enrichment Analysis (GSEA) indicated a significant enrichment of NF-κB target genes (http://www.bu.edu/nf-kb/gene-resources/target-genes) in the down-regulated genes of the datasets for RACA, JΔM-CA and JΔM-RA cells at 3 and 18 hrs of stimulation (FDR<0.001) (Figure 7D and Table S4). Ingenuity Pathway Analysis (Ingenuity Systems) linked the signatures of the RACA, JΔM-CA and JΔM-RA cells with reduced activation/proliferation of lymphocytes and inhibition of NF-κB signalling (Table S5). Collectively, these data demonstrate that MALT1 auto-processing is required to induce optimal transcription of NF-κB target genes in activated Jurkat T cells.

## Discussion

Here, we provide several lines of evidence for an essential role of MALT1 auto-proteolysis in NF-κB dependent gene transcription in activated lymphocytes. First, activation of MALT1 induced its proteolytic cleavage at R149 in 293T cells. Second, recombinant MALT1 was able to cleave itself *in vitro* at R149. Third, continuous MALT1 auto-proteolysis was observed in ABC-DLBCL cells and SSK41 MALT lymphoma cells that have constitutive MALT1 protease activity. Fourth, B- and T-cell stimulation induced MALT1 cleavage. Finally, an un-cleavable MALT1 mutant did not prevent initial IκBα phosphorylation and nuclear accumulation of NF-κB subunits but impaired the transcriptional activation of NF-κB target genes.

TCR engagement induces the redistribution of BCL10 and MALT1 to the membrane rafts at the TCR complex, which is essential to activate NF-κB signalling [22], [23], [51]. Artificial membrane anchoring of MALT1 not only activated its protease activity and NF-κB signalling, but also induced MALT1 auto-proteolysis. The resulting p76 cleavage product efficiently oligomerized and activated NF-κB signalling in a TRAF6-dependent manner. TRAF6 mediates K63-linked poly-ubiquitination of itself, MALT1 and IKKγ which facilitates activation of the IKK complex and phosphorylation of IκBα. This mechanism and also the nuclear accumulation of the NF-κB subunits were however not affected in MALT1-deficient Jurkat T cells expressing un-cleavable MALT1 (JΔM-RA), suggesting a role for MALT1 auto-proteolysis further downstream in regulating NF-κB transcriptional activation. Interestingly, MALT1 was reported to shuttle between the nucleus and cytoplasm and its nuclear retention reduced NF-κB signalling [52], suggesting an inhibitory function for MALT1 in the nucleus. Whether MALT1 auto-proteolysis relieves this inhibitory potential on NF-κB signalling will be an interesting aspect of future work.

**Figure 8:**
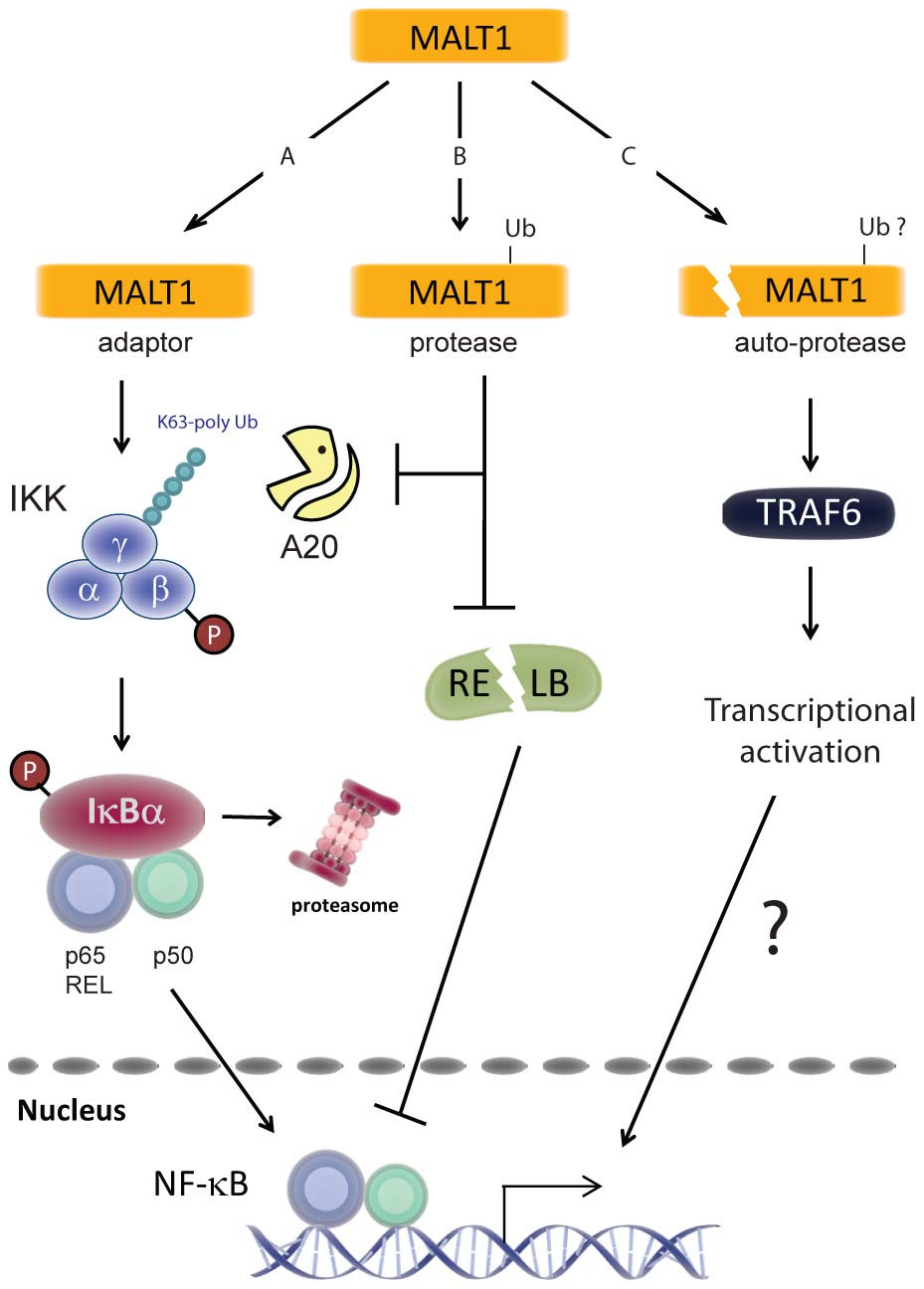
Model for MALT1 functions in TCR-mediated NF-κB1 activation. A) The adaptor function of MALT1 is required for TCR-mediated activation of the IKK complex. Via formation of the CARMA1/BCL10/MALT1 complex MALT1 controls TRAF6-mediated K63 poly-ubiquitination of the gamma subunit of the IKK complex. Concurrent phosphorylation of IKKβ activates the IKK complex that phosphorylates the NF-κB inhibitor IκB, induces its proteasomal degradation and allows nuclear translocation of NF-κB complexes consisting of p50, p65 and REL. B) Parallel induction of MALT1 protease activity prevents de-ubiquitination of IKKγ and possibly other substrates via A20 cleavage and facilitates DNA binding of p65- or REL-containing NF-κB complexes via RELB cleavage. C) MALT1 auto-proteolysis represents a third level of MALT1 regulation that controls in a TRAF6-dependent and BCL10-independent manner the transcriptional activation of nuclear NF-κB complexes via a yet unknown mechanism.

MALT1 controls T- and B-cell activation via both its adaptor and protease function. As an adaptor, MALT1 is required for building up the proximal signalling complex that controls the IKK-dependent activation of the canonical NF-κB pathway, as well as the activation of the c-JUN N-terminal kinase (JNK) dependent transcriptional pathway. The protease function of MALT1 apparently serves to promote gene transcription by inactivating negative regulators of NF-κB and JNK signalling, like A20, RELB and CYLD. Moreover, MALT1-dependent cleavage of the RNAse MCPIP1 (also known as Regnase-1) is thought to lead to the stabilization of the resulting transcripts [35]. Auto-processing of MALT1 did not affect these functions, since the processing-deficient R149A mutant showed normal protease activity and an unaltered capacity to promote IKK or JNK activation. The data presented in this study therefore reveal a highly interesting novel aspect of MALT1's function, which is controlled by the auto-proteolytic removal of the N-terminal death domain and the BCL10 binding site. This results in the formation of an active C-terminal p76 fragment of MALT1 that dissociates from BCL10 and oligomerizes to promote NF-κB-dependent transcription in a TRAF6-dependent manner. These findings support a model in which the p76 fragment of MALT1, in combination with TRAF6 and potentially additional components, directly or indirectly affects the transcriptional activity of NF-κB complexes by means that remain to be discovered (Figure 8).

In conclusion, our study identifies MALT1 auto-proteolysis as essential for optimal NF-κB transcriptional activity in antigen receptor signalling and further strengthens the position of MALT1 protease as an attractive target for immune-suppression.

## Materials and Methods

### Antibodies and plasmids

Primary antibodies used in this study included antibodies specific for the Flag epitope (M2), β-Actin (A1978) and tubulin (B-5–1-2) from Sigma-Aldrich, HA (12CA5) from Roche, A20 (ab45366) and MALT1-N (ab33921) from Abcam, BCL10 (sc-5273 and sc-5611), TRAF6 (sc-7221), CYLD E10 (sc-74435), p65 (sc-372), p50 (sc-114), c-Rel/REL (sc-71), Lck (sc-433) and cJun-P (sc-16312) from Santa Cruz Biotechnology, and P-IκB-α (Ser32/36, 5A5), P-ERK (Thr202/Tyr204, #9101), P-p38 (#9211), P-JNK (#9255), RelB (#4922) and NIK (#4994) from Cell Signaling Technology and GAPDH (MAB374) from Millipore. Anti-MALT1-C [5], anti-MALT1 [31] and anti-cleaved BCL10 [38] were reported previously. Anti-p76 is an affinity purified rabbit polyclonal raised against a nonapeptide (ATGHPFVQY) corresponding to the N-terminus of cleaved MALT1 p76 (Eurogentec).

All constructs for expressing proteins in eukaryotic cells were made in pcDNA3.1 (Clonetech Laboratories) encoding an N-terminal Flag- (with or without a StrepII-tag) or HA-epitope or in pCR3 (InVitrogen, constructs used for Figure 2A). To direct raft association of MALT1, the myristoylation/palmitoylation (mp) motif of Lck (MGCVCSSNPEDD) was inserted in front of the Flag epitope (pcD-mp-F-MALT1). Vectors enabling expression of biotinylated proteins were described previously [45]. For expression of native p76, its coding sequence was cloned in frame with HA-tagged ubiquitin at the N-terminus and the Avi-tag at the C-terminus in pcDNA3.1.

### Cell Culture

HEK293T (or 293T) (ATCC CRL-11268), HKB11 cells (ATCC CRL-12568) and SSK41 ([53], kindly provided by Dr. M Dyer) cells were cultured in DMEM-F12 (Life Technologies) supplemented with 10% fetal calf serum (FCS). Jurkat T cells [54], RAJI B cells (ATCC CCL-86) and the DLBCL cell lines BJAB [55], HBL-1 [56] and OCI-Ly3 [57] were grown in RPMI medium 1640 (Life technologies) supplemented with antibiotics and 10% FCS (for Jurkat, RAJI, and BJAB) or 20% FCS (HBL-1 and OCI-Ly3). Jurkat T cells with stable expression of MALT1, MALT1-C464A, MALT1-R149A or the double mutant MALT1-RACA were generated as described [30].

For stimulation of cells, a mixture of PMA (phorbol 12-myristate 13-acetate; 10-50 ng/ml; Alexis Biochemicals) and ionomycin (1 µM; Calbiochem) was used. In some experiments, cells were pre-incubated with 50 µM z-VRPR-fmk (Bachem AG) for 30 min before stimulation with PMA and ionomycin. DLBCL cell lines and SSK41 MALT1 lymphoma cells were treated with z-VRPR-fmk or z-LVSR-fmk (Bachem AG) for 36 hrs.

### MALT1 *in vitro* assay

HKB11 cells were transfected (TurboFect, Fermentas) with a linearized expression construct for Flag-StrepII-tag-MALT1 (F-STII-MALT1) in a pcDNA3.1-puro vector and clones with stable expression were obtained after limiting dilution. Clones with high expression were adapted for high-density suspension culture in 293 serum free medium II (293 SFM II, Life Technologies). Recombinant MALT1 was purified using Strep-Tactin Superflow resin according to the manufacturer's protocol (IBA). Purified F-STII-MALT1 (1 µg) was incubated at 37°C for 2 h in a total volume of 25 µl paracaspase assay buffer (50 mM MES, pH 6.8, 150 mM NaCl, 10% (wt/vol) sucrose, 0.1% (wt/vol) CHAPS (3-[(3-cholamidopropyl)-dimethylammonio]-1-propane sulfonate hydrate), 10 mM dithiothreitol and 0.2, 0.4, 0.6 or 0.8 M NH_4_-citrate respectively, with or without MALT1 protease inhibitors z-VRPR-fmk (Bachem AG) and z-LVSR-fmk (Bachem AG). An aliquot of the reactions was diluted in (1/100) in paracaspase assay buffer containing 50 µM Ac-LVSR-AMC (Genecust) to measure MALT1 protease activity. 25 µl reactions in 384-well black plates (Greiner Bio One) were measured with excitation/emission wavelengths of 390/460 in a plate reader (BMG Labtech).

Both wild-type human MALT1 (S2-K824) and a protease inactivated MALT1 (S2-K824) with a C464A mutation was fused with the leucine-zipper sequence from GCN4 (251–281) in the N-terminus (LZ-MALT1) as described [47]. On the N-terminal preceding the LZ-tag an Avi-tag and a 6xHisAsn-tag was added to the constructs that was cloned into pFastBac1, followed by virus generation and protein expression in S. frugiperda (Sf21) [58]. LZ-MALT1 was purified by Ni-NTA affinity chromatography (Qiagen) followed by gel filtration chromatography on a Superdex 200 16/60 (GE Healthcare) in buffer containing 25 mM Tris-Cl (pH 7.5), 150 mM NaCl, 10% (w/v) glycerol and 1 mM TCEP. MALT1 protease *in vitro* reactions were performed as described for F-STII-MALT1.

### FRET-based and Western blot analysis of MALT1 protease activity

FRET-based determination of MALT1 activity in 293T cells was essentially done as described (41). In brief, cells were transfected with the eYFP–Leu-Val-Ser-Arg–eCFP reporter construct, together with indicated combinations of MALT1 and BCL10 expression constructs. 24 h after transfection, cells were resuspended in flow cytometry buffer (1% FCS and 1 mM EDTA in PBS) and analyzed with an LSR II (BD Biosciences) containing 405-, 488-, 561- and 640-nm lasers. To measure the eCFP and FRET signal, the transfected cells were excited with a standard 450/50 filter for collection of the eCFP fluorescence and a 585/42 filter for FRET fluorescence, respectively, and for each sample at least 5,000 highly eYFP+ cells were counted (41). In parallel to flow cytometry, cell lysates were assessed for eYFP–Leu-Val-Ser-Arg–eCFP reporter cleavage by Western blot using anti-GFP (ALX 210-199; Enzo LifeSciences).

### TALEN-mediated targeted disruption of MALT1 in Jurkat T cells

TALENs that target a BfaI site (ttctttctgttgctttcAGTTGCCTAGACCTGgagcagtgttctcttaa) in the 5′ end of exon2 of MALT1 were constructed by Cellectis. Jurkat clones expressing MALT1-C464A and MALT1-R149A were electroporated as above with the TALENs followed by single cell dilution and expansion in 96 well plates. Cells were lysed in 400mM KOH/100mM DTT for 5′ at 4°C, sample was freeze/thawed (2X), the lysate was neutralized with an equal volume of 400mM HCL/600mM Tris pH7.5, and then used directly for PCR amplification (primers in Table S1). PCR products for MALT1 intron1-exon2 were digested with BfaI to identify clones with a deletion/mutation in exon2, which were confirmed by sequencing individual cloned PCR fragments (pGEM-T-easy, Promega). The procedure was repeated to inactivate and confirm deletion of the second MALT1 allele. Bi-allelic MALT1 inactivation was confirmed for two clones of MALT1-C464A (JΔM –CA1 and -CA2) and two of MALT1-R149A (JΔM –RA1 and -RA2) by sequence analysis of larger PCR fragments that contain a SNP located 489 bp upstream of exon 2 of MALT1 (Figure S6). The same procedure was applied to generate Jurkat T cells deficient for MALT1 (JΔM).

### Western Blot Analysis, bio-IPs and purification of Detergent Resistant Membrane (DRM) fractions

For some experiments (Figures 5A/B/C and 6A), cells were lysed in ice-cold HEPES-NaCl lysis buffer (50 mM HEPES, pH 7.4, 150 mM NaCl, and 1% Triton X-100) complemented with inhibitors of proteases (0.2 mg/mL AEBSF, 2 µg/mL aprotinin, and 10 µg/mL leupeptin) and phosphatases (50 mM NaF, 10 mM Na4P2O7, and 10 mM NaVO4). Postnuclear cell lysates were boiled with reducing SDS sample buffer and analysed by SDS-PAGE on 15% gels.

Avi-tagged proteins become biotinylated in eukaryotic cells via co-expression of the *E. coli* BirA biotin protein ligase [59]. After washing with PBS, cells were lysed for 30 min on ice in non-denaturing lysis buffer (NDLB: 20 mM Tris-Cl pH 7,6, 110 mM NaCl, 2 mM EDTA, 0,3% NP-40 and 10% glycerol, Supplemented with phosphatase inhibitors (30 mM NaF, 1 mM Na_3_VO_4_, 2 mM Na_2_MoO_4_, 5 mM Na_4_P_2_O_7_) and 1X Complete protease inhibitor cocktail (Roche). The biotinylated protein complex is precipitated using paramagnetic streptavidin beads (Dynabeads M-280, Invitrogen) for 1 hour at 4°C. Protein precipitates were washed four times in lysis buffer and boiled for 10 min with reducing SDS sample buffer. All samples were size separated on 4-12% SDS-polyacrylamide gels (NuPage, Invitrogen) and transferred to polyvinylidene difluoride membranes (GE Healthcare) for detection. Lipid raft purifications were performed as described previously [5].

### NF-κB Reporter Assays, ELISA and FRET assay

NF-κB reporter assays in 293T cells were performed as described [5]. Nucleofection of Jurkat T cells was performed according to the manufacturer's recommendations (Amaxa Cell Line Nucleofector Kit V, Lonza AG). IL-2 and CSF2 in the supernatant of the different Jurkat clones, unstimulated or stimulated for 18 hrs with 75 ng/ml PMA – 150 ng/ml ionomycin, was measured by ELISA (OptEIA hIL-2 ELISA kit, BD Pharmingen) according to the manufacturer's protocol. For the cellular MALT1 protease assay, HEK293T cells were transfected the eYFP–LVSR–eCFP probe and MALT1/BCL10 constructs, and analysed by flow cytometry for gain of eCFP fluorescence as described [41].

### Quantitative RT-PCR and RNA sequencing

RNA isolation and cDNA synthesis were performed using standard protocols. Quantitative RT-PCR was performed with the LightCycler 480 SYBR Green I master mix (Roche Diagnostics) and analyzed using the comparative dCt method using HPRT1 as a reference control. Primer sequences are shown in the Table S1.

For RNA sequencing, the libraries were prepared according to the standard Illumina TruSeq RNA sample preparation protocol (Illumina). RNAseq libraries were constructed for Jurkat T cells expressing MALT1 (2X), MALT1 RACA (2X) and for JΔM-CA1, JΔM-CA2, JΔM-RA1, JΔM-RA2 (each 1X), and this un-stimulated or after stimulation with 75 ng/ml PMA – 150 ng/ml ionomycin for 3 and 18 hrs respectively. Each library was sequenced on an Illumina HISeq 2000 according to the manufacturer's recommendations generating single-end 50 bp reads. Differential gene expression between RNA-sequencing datasets was analyzed using TopHat and Cufflinks as described (Trapnell *et al.*, 2012), using JΔM-CA1/JΔM-CA2 and JΔM-RA1/JΔM-RA2 as experimental repeats. The RNA sequencing data have been submitted to the GEO database and assigned the accession number GSE52934.

### Functional and pathway analysis of RNAseq data

Ingenuity Pathway Analysis (Ingenuity Systems) was used for biological knowledge mining. Enrichment tests with a gene set for NF-kB targets (http://www.bu.edu/nf-kb/gene-resources/target-genes) was carried out using the Gene Set Enrichment Analysis (GSEA) software. The q values for each gene-set were calculated by using 1000 permutations and a False Discovery Rate <25%.

## Supporting Information

Figure S1 **MALT1 cleavage does not affect its protease activity.** A) Immunoblot of lysates of 293T cells transiently expressing mp-MALT1 and its mutants as specified with a-MALT1-N. eMALT1: endogenous MALT1. Arrow indicates the N-terminal p19 cleavage fragment. All molecular mass standards are in kDa. B) Immunoblot of lysates of 293T cells transiently expressing mp-MALT1, its R149A and C464A mutants, and Ubiquitin-p76 with indicated antibodies. Arrows indicate the MALT1 (p76), CYLD (p70) and A20 (p50) cleavage fragments respectively. * non-specific fragments. LC: a-specific fragment detected with the p76 neo-epitope antibody used as loading control. C) HEK293T cells were transfected with the eYFP–Leu-Val-Ser-Arg–eCFP probe (eYFP-LVSR-eCFP) and the indicated constructs. Probe cleavage (as gain in eCFP fluorescence, labeled as “% of fluorescent cells with FRET loss” in the y-axis of the graph) was assessed by flow cytometry, gating on eYFPhi cells (upper panel). In addition, cell lysates were analyzed by blotting for MALT1, BCL10, GFP and Tubulin, as indicated (lower panel). Compared to flow cytometry, in which only eYFPhi cells are included in the analysis, the Western blot analysis shows a higher percentage of reporter cleavage because all cells (including cells expressing low levels of the reporter) are lysed and analyzed.
(TIF)

Figure S2 **MALT1 and API2-MALT1 autoproteolysis in SSK41 lymphoma cells.** A) Immunoblot of lysates from SSK41 cells, left untreated or treated with 50 µM z-VRPR-fmk (36h), with antibodies against MALT1, cleaved BCL10 and tubulin. Arrow indicates the MALT1 p19 cleavage fragment. B) Features of the A7M3 fusion variant of API2-MALT1 plus the domain content (solid bars) of the 53 and 76 kDa cleavage fragments. BIR: Baculovirus “inhibition of apoptosis” repeat. C) Immunoblot analysis of lysates of 293T cells transiently expressing increasing concentrations of Flag-tagged A7M3 and A7M3-R149A mutant, indicating the p76 C-terminal fragment detected with a-MALT1-C (left) or the p76 neo-epitope specific antibody (middle). Right: Immunoblot analysis of 293T cells transiently expressing the API2-MALT1 fusion variant A7M3, A7M3-R149A and A7M3-C464A with antibodies against CYLD and A20. Arrows indicate their respective p70 and p50 cleavage fragments. Immunoblot with the Flag antibody (N-terminus) was performed to demonstrate equal expression of A7M3 or its mutants and shows the N-terminal cleavage fragment (p54) of A7M3. * non-specific fragment.
(TIF)

Figure S3 **MALT1 undergoes auto-proteolysis** ***in vitro*** **.** Top: *In vitro* cleavage of the fluorogenic tetrapeptide substrate Ac-LVSR-AMC (50 µM) by F-STII-MALT1 in increasing concentrations of the cosmotropic salt NH4-citrate (0.2, 0.4, 0.6, and 0.8 M). The barchart shows cleavage activity as Fluorescence Units (FU) increase/min. Results are expressed as means ± SD (n = 3). Bottom: enzymatic reactions were analysed by immunoblotting with a-MALT1-N. The blot was previously cut in two to detect p76 and p19 separately, which explains the white line in the middle.
(TIF)

Figure S4 **MALT1 auto-proteolysis is not required for initial IκBα phosphorylation and NF-κB nuclear translocation in Jurkat T cells overexpressing MALT1 mutants.** Jurkat T cells expressing MALT1 or the mutants C464A, R149A and RACA were stimulated with P/I for indicated times and cytosolic and nuclear extracts were immunoblotted with indicated antibodies. Blots used to detect c-Rel were re-used without stripping to detect RELB and therefore both bands are visible in the RELB panel (upper band  =  c-Rel, lower band  =  RELB).
(TIF)

Figure S5 **MALT1 auto-proteolysis is not required for initial IκBα phosphorylation and NF-κB nuclear translocation in JΔM-CA and JΔM-RA cells.** A) Jurkat T cells expressing MALT1-C464A or MALT1-R149A were genetically modified with TALENs to inactivate endogenous MALT1 expression generating JΔM-CA and JΔM-RA cells respectively. Cells were stimulated with P/I for indicated times and cytosolic and nuclear extracts were immunoblotted with indicated antibodies. LC: a-specific band used as loading control. B) JΔM-CA and JΔM-RA cells were pre-treated with MG-132 for 30 min before stimulation for 15 or 30 min with PMA/ionomycin (P/I). Total cell lysates were immunoblotted with indicated antibodies. LC: a-specific band used as loading control. C) Immunoblot with a-MALT1-N showing expression of ectopic MALT1 and mutants relative to endogenous MALT1 (lane 5) in the different Jurkat cell lines. β-actin: loading control.
(TIF)

Figure S6 **TALEN-mediated knock-out of endogenous MALT1.** Jurkat T cells and Jurkat T cells with ectopic expression of MALT1-R149A and MALT1-C464A were electroporated with TALEN pairs targeting a BfaI at the intron1-exon2 boundary of MALT1. Position and size of the introduced deletions in the different generated cell lines are indicated. A single nucleotide polymorphism located 489 bp upstream of exon 2 of MALT1 was used to discriminate the 2 MALT1 alleles.
(TIF)

Table S1 **Sequences of primer pairs used for qRT-PCR analysis.**
(XLSX)

Table S2 **Differentially expressed genes at 3 and 18 hrs of stimulation with PMA/Ionomycin in JΔM-CA, JΔM-RA and RACA vs MALT1 expressing cells with more than 2 fold change and FDR q<0,001.**
(XLSX)

Table S3 **qRT-PCR validation of differentially expressed genes between MALT1 and JΔM-CA, JΔM-RA and RACA respectively at 3 and 18 hrs of stimulation with PMA/Ionomycin.**
(XLSX)

Table S4 **Gene set enrichment analysis of NF-κB target genes (Boston) in the pre-ranked sets of “differentially expressed genes” for JΔM-CA, JΔM-RA and RACA at 3 and 18 hrs of stimulation with P/I.**
(XLSX)

Table S5 **Ingenuity Pathway analysis of differential expression of JΔM-CA, JΔM-RA and RACA cells.**
(XLSX)

## References

[1] Dierlamm J, Baens M, Wlodarska I, Stefanova-Ouzounova M, Hernandez JM, et al (1999) The apoptosis inhibitor gene API2 and a novel 18q gene, MLT, are recurrently rearranged in the t(11;18)(q21;q21) associated with mucosa- associated lymphoid tissue lymphomas. Blood 93: 3601–3609. 10339464

[2] Lucas PC, Yonezumi M, Inohara N, McAllister-Lucas LM, Abazeed ME, et al (2001) Bcl10 and MALT1, independent targets of chromosomal translocation in MALT lymphoma,cooperate in a novel NF-kappaB signaling pathway. J Biol Chem 276: 19012–19019. 11262391 10.1074/jbc.M009984200

[3] Uren GA, O'Rourke K, Aravind L, Pisabarro TM, Seshagiri S, et al (2000) Identification of paracaspases and metacaspases: two ancient families of caspase-like proteins, one of which plays a key role in MALT lymphoma. Mol Cell 6: 961–967. 11090634 10.1016/s1097-2765(00)00094-0

[4] Zhou HL, Du MQ, Dixit VM (2005) Constitutive NF-kappa B activation by the t(11;18)(q21;q21) product in MALT lymphoma is linked to deregulated ubiquitin ligase activity. Cancer Cell 7: 425–431. 15894263 10.1016/j.ccr.2005.04.012

[5] Baens M, Fevery S, Sagaert X, Noels H, Hagens S, et al (2006) Selective Expansion of Marginal Zone B-Cells in Eµ-API2-MALT1 Mice is Linked to Enhanced IκB kinase γ Polyubiquitination. Cancer Res 66: 5270–5277. 16707452 10.1158/0008-5472.CAN-05-4590

[6] Willis TG, Jadayel DM, Du MQ, Peng H, Perry AR, et al (1999) Bcl10 is involved in t(1;14)(p22;q32) of MALT B cell lymphoma and mutated in multiple tumor types. Cell 96: 35–45. 9989495 10.1016/s0092-8674(00)80957-5

[7] Zhang Q, Siebert R, Yan M, Hinzmann B, Cui X, et al (1999) Inactivating mutations and overexpression of BCL10, a caspase recruitment domain-containing gene, in MALT lymphoma with t(1;14)(p22;q32). Nat Genet 22: 63–68. 10319863 10.1038/8767

[8] Sanchez-Izquierdo D, Buchonnet G, Siebert R, Gascoyne RD, Climent J, et al (2003) MALT1 is deregulated by both chromosomal translocation and amplification in B-cell non-Hodgkin lymphoma. Blood 101: 4539–4546. 12560219 10.1182/blood-2002-10-3236

[9] Streubel A, Lamprecht A, Dierlamm J, Cerroni L, Stolte M, et al (2003) t(14;18)(q32;q21) involving IGH and MALT1 is a frequent chromosomal aberration in MALT lymphoma. Blood 101: 2335–2339. 12406890 10.1182/blood-2002-09-2963

[10] Thome M (2008) Multifunctional roles for MALT1 in T-cell activation. Nat Rev Immunol 8: 495–500. 18575460 10.1038/nri2338

[11] Ruland J, Duncan GS, Elia A, del Barco Barrantes I, Nguyen L, et al (2001) Bcl10 is a positive regulator of antigen receptor-induced activation of NF-kappaB and neural tube closure. Cell 104: 33–42. 11163238 10.1016/s0092-8674(01)00189-1

[12] Ruland J, Duncan GS, Wakeham A, Mak TW (2003) Differential Requirement for Malt1 in T and B Cell Antigen Receptor Signaling. Immunity 19: 749–758. 14614861 10.1016/s1074-7613(03)00293-0

[13] Ruefli-Brasse AA, French DM, Dixit VM (2003) Regulation of NF-kappa B-dependent lymphocyte activation and development by paracaspase. Science 302: 1581–1584. 14576442 10.1126/science.1090769

[14] Egawa T, Albrecht B, Favier B, Sunshine MJ, Mirchandani K, et al.. (2003) Requirement for CARMA1 in antigen receptor-induced NF-kappa B activation and lymphocyte proliferation. Curr Biol 13: 1252–1258. S0960982203004913 [pii]. 10.1016/s0960-9822(03)00491-3 12867038

[15] Jun JE, Wilson LE, Vinuesa CG, Lesage S, Blery M, et al (2003) Identifying the MAGUK protein CARMA-1 as a central regulator of humoral immune responses and atopy by genome-wide mouse mutagenesis. Immunity 18: 751–762. 12818157 10.1016/s1074-7613(03)00141-9

[16] Hara H, Wada T, Bakal C, Kozieradzki I, Suzuki S, et al (2003) The MAGUK family protein CARD11 is essential for lymphocyte activation. Immunity 18: 763–775. 12818158 10.1016/s1074-7613(03)00148-1

[17] Newton K, Dixit VM (2003) Mice lacking the CARD of CARMA1 exhibit defective B lymphocyte development and impaired proliferation of their B and T lymphocytes. Curr Biol 13: 1247–1251. S0960982203004585 [pii]. 10.1016/s0960-9822(03)00458-5 12867037

[18] Sommer K, Guo B, Pomerantz JL, Bandaranayake AD, Moreno-Garcia ME, et al (2005) Phosphorylation of the CARMA1 linker controls NF-kappaB activation. Immunity 23: 561–574. 16356855 10.1016/j.immuni.2005.09.014

[19] Matsumoto R, Wang D, Blonska M, Li H, Kobayashi M, et al (2005) Phosphorylation of CARMA1 plays a critical role in T Cell receptor-mediated NF-kappaB activation. Immunity 23: 575–585. 16356856 10.1016/j.immuni.2005.10.007

[20] Kabouridis PS (2006) Lipid rafts in T cell receptor signalling. Mol Membr Biol 23: 49–57. 16611580 10.1080/09687860500453673 PMC2596298

[21] Wang D, You Y, Case SM, McAllister-Lucas LM, Wang L, et al (2002) A requirement for CARMA1 in TCR-induced NF-kappa B activation. Nat Immunol 3: 830–835. 12154356 10.1038/ni824

[22] Gaide O, Favier B, Legler DF, Bonnet D, Brissoni B, et al (2002) CARMA1 is a critical lipid raft-associated regulator of TCR-induced NF-kappa B activation. Nat Immunol 3: 836–843. 12154360 10.1038/ni830

[23] Che TJ, You Y, Wang DH, Tanner MJ, Dixit VM, et al (2004) MALT1/paracaspase is a signaling component downstream of CARMA1 and mediates T cell receptor-induced NF-kappa B activation. J Biol Chem 279: 15870–15876. 14754896 10.1074/jbc.M310599200

[24] Qiao Q, Yang C, Zheng C, Fontan L, David L, et al (2013) Structural architecture of the CARMA1/Bcl10/MALT1 signalosome: nucleation-induced filamentous assembly. Mol Cell 51: 766–779 S1097-2765(13)00632-1 [pii]; 10.1016/j.molcel.2013.08.032 [doi] 24074955 PMC3929958

[25] Oeckinghaus A, Wegener E, Welteke V, Ferch U, Arslan SC, et al (2007) Malt1 ubiquitination triggers NF-kappaB signaling upon T-cell activation. EMBO J 26: 4634–4645 7601897 [pii]; 10.1038/sj.emboj.7601897 [doi] 17948050 PMC2080808

[26] Wu CJ, Ashwell JD (2008) NEMO recognition of ubiquitinated Bcl10 is required for T cell receptor-mediated NF-kappaB activation. Proc Natl Acad Sci U S A 105: 3023–3028. 18287044 10.1073/pnas.0712313105 PMC2268578

[27] Sun LJ, Deng L, Ea CK, Xia ZP, Chen ZJJ (2004) The TRAF6 ubiquitin ligase and TAK1 kinase mediate IKK activation by BCL10 and MALT1 in T lymphocytes. Mol Cell 14: 289–301. 15125833 10.1016/s1097-2765(04)00236-9

[28] Wu CJ, Conze DB, Li T, Srinivasula SM, Ashwell JD (2006) NEMO is a sensor of Lys 63-linked polyubiquitination and functions in NF-kappaB activation. Nat Cell Biol 8: 398–406. 16547522 10.1038/ncb1384

[29] Zhou HL, Wertz I, O'Rourke K, Ultsch M, Seshagiri S, et al (2004) Bcl10 activates the NF-kappa B pathway through ubiquitination of NEMO. Nature 427: 167–171. 14695475 10.1038/nature02273

[30] Coornaert B, Baens M, Heyninck K, Bekaert T, Haegman M, et al (2008) T cell antigen receptor stimulation induces MALT1 paracaspase-mediated cleavage of the NF-kappaB inhibitor A20. Nat Immunol 9: 263–271. 18223652 10.1038/ni1561

[31] Rebeaud F, Hailfinger S, Posevitz-Fejfar A, Tapernoux M, Moser R, et al (2008) The proteolytic activity of the paracaspase MALT1 is key in T cell activation. Nat Immunol 9: 272–281. 18264101 10.1038/ni1568

[32] Weih F, Lira SA, Bravo R (1996) Overexpression of RelB in transgenic mice does not affect I kappa B alpha levels: differential regulation of RelA and RelB by the inhibitor protein. Oncogene 12: 445–449. 8570223

[33] Hailfinger S, Nogai H, Pelzer C, Jaworski M, Cabalzar K, et al (2011) Malt1-dependent RelB cleavage promotes canonical NF-kappaB activation in lymphocytes and lymphoma cell lines. Proc Natl Acad Sci U S A 108: 14596–14601 1105020108 [pii]; 10.1073/pnas.1105020108 [doi] 21873235 PMC3167514

[34] Staal J, Driege Y, Bekaert T, Demeyer A, Muyllaert D, et al (2011) T-cell receptor-induced JNK activation requires proteolytic inactivation of CYLD by MALT1. EMBO J 30: 1742–1752 emboj201185 [pii];10.1038/emboj.2011.85 [doi] 21448133 10.1038/emboj.2011.85 PMC3101995

[35] Uehata T, Iwasaki H, Vandenbon A, Matsushita K, Hernandez-Cuellar E, et al (2013) Malt1-induced cleavage of regnase-1 in CD4(+) helper T cells regulates immune activation. Cell 153: 1036–1049 S0092-8674(13)00510-2 [pii]; 10.1016/j.cell.2013.04.034 [doi] 23706741

[36] Rosebeck S, Madden L, Jin X, Gu S, Apel IJ, et al (2011) Cleavage of NIK by the API2-MALT1 fusion oncoprotein leads to noncanonical NF-kappaB activation. Science 331: 468–472 331/6016/468 [pii]; 10.1126/science.1198946 [doi] 21273489 PMC3124150

[37] Ferch U, Kloo B, Gewies A, Pfander V, Duwel M, et al (2009) Inhibition of MALT1 protease activity is selectively toxic for activated B cell-like diffuse large B cell lymphoma cells. J Exp Med 206: 2313–2320 jem.20091167 [pii]; 10.1084/jem.20091167 [doi] 19841089 PMC2768866

[38] Hailfinger S, Lenz G, Ngo V, Posvitz-Fejfar A, Rebeaud F, et al (2009) Essential role of MALT1 protease activity in activated B cell-like diffuse large B-cell lymphoma. Proc Natl Acad Sci U S A 106: 19946–19951 0907511106 [pii]; 10.1073/pnas.0907511106 [doi] 19897720 PMC2785272

[39] Ngo VN, Davis RE, Lamy L, YU X, Zhao H, et al (2006) A loss-of-function RNA interference screen for molecular targets in cancer. Nature 441: 106–110. 16572121 10.1038/nature04687

[40] Boatright KM, Renatus M, Scott FL, Sperandio S, Shin H, et al (2003) A unified model for apical caspase activation. Mol Cell 11: 529–541. 12620239 10.1016/s1097-2765(03)00051-0

[41] Pelzer C, Cabalzar K, Wolf A, Gonzalez M, Lenz G, et al (2013) The protease activity of the paracaspase MALT1 is controlled by monoubiquitination. Nat Immunol 14: 337–345 ni.2540 [pii]; 10.1038/ni.2540 [doi] 23416615

[42] Cabalzar K, Pelzer C, Wolf A, Lenz G, Iwaszkiewicz J, et al (2013) Monoubiquitination and activity of the paracaspase MALT1 requires glutamate 549 in the dimerization interface. PLoS ONE 8: e72051 10.1371/journal.pone.0072051 [doi];PONE-D-13-18063 [pii] 23977204 PMC3747146

[43] Hara H, Bakal C, Wada T, Bouchard D, Rottapel R, et al (2004) The molecular adapter Carma1 controls entry of IkappaB kinase into the central immune synapse. J Exp Med 200: 1167–1177. 15520247 10.1084/jem.20032246 PMC2211862

[44] Resh MD (1999) Fatty acylation of proteins: new insights into membrane targeting of myristoylated and palmitoylated proteins. Biochim Biophys Acta 1451: 1–16. S0167-4889(99)00075-0 [pii]. 10.1016/s0167-4889(99)00075-0 10446384

[45] Noels H, Van Loo G, Hagens S, Broeckx V, Beyaert R, et al (2007) A novel TRAF6 binding site in MALT1 defines distinct mechanisms of NF-kappa B activation by API2-MALT1 fusions. J Biol Chem 282: 10180–10189. 17287209 10.1074/jbc.M611038200

[46] Hachmann J, Snipas SJ, van Raam BJ, Cancino EM, Houlihan EJ, et al (2012) Mechanism and specificity of the human paracaspase MALT1. Biochem J 443: 287–295 BJ20120035 [pii]; 10.1042/BJ20120035 [doi] 22309193 PMC3304489

[47] Fontan L, Yang C, Kabaleeswaran V, Volpon L, Osborne MJ, et al (2012) MALT1 small molecule inhibitors specifically suppress ABC-DLBCL in vitro and in vivo. Cancer Cell 22: 812–824 S1535-6108(12)00483-7 [pii]; 10.1016/j.ccr.2012.11.003 [doi] 23238016 PMC3984478

[48] Qiu L, Dhe-Paganon S (2011) Oligomeric Structure of the MALT1 Tandem Ig-Like Domains. PLoS ONE 6: e23220 10.1371/journal.pone.0023220 [doi];PONE-D-10-03906 [pii] 21966355 PMC3179463

[49] Davis RE, Brown KD, Siebenlist U, Staudt LM (2001) Constitutive nuclear factor kappaB activity is required for survival of activated B cell-like diffuse large B cell lymphoma cells. J Exp Med 194: 1861–1874. 11748286 10.1084/jem.194.12.1861 PMC2193582

[50] Lenz G, Davis RE, Ngo VN, Lam L, George TC, et al (2008) Oncogenic CARD11 mutations in human diffuse large B cell lymphoma. Science 319: 1676–1679. 18323416 10.1126/science.1153629

[51] Wang D, Matsumoto R, You Y, Che T, Lin XY, et al (2004) CD3/CD28 costimulation-induced NF-kappaB activation is mediated by recruitment of protein kinase C-theta, Bcl10, and IkappaB kinase beta to the immunological synapse through CARMA1. Mol Cell Biol 24: 164–171. 14673152 10.1128/MCB.24.1.164-171.2003 PMC303359

[52] Nakagawa M, Hosokawa Y, Yonezumi M, Izumiyama K, Suzuki R, et al (2005) MALT1 contains nuclear export signals and regulates cytoplasmic localization of BCL10. Blood 106: 4210–4216. 16123224 10.1182/blood-2004-12-4785

[53] Sideras P, Mizuta TR, Kanamori H, Suzuki N, Okamoto M, et al (1989) Production of sterile transcripts of C gamma genes in an IgM-producing human neoplastic B cell line that switches to IgG-producing cells. Int Immunol 1: 631–642. 2518730 10.1093/intimm/1.6.631

[54] Schneider U, Schwenk HU, Bornkamm G (1977) Characterization of EBV-genome negative "null" and "T" cell lines derived from children with acute lymphoblastic leukemia and leukemic transformed non-Hodgkin lymphoma. Int J Cancer 19: 621–626. 68013 10.1002/ijc.2910190505

[55] Klein G, Lindahl T, Jondal M, Leibold W, Menezes J, et al (1974) Continuous lymphoid cell lines with characteristics of B cells (bone-marrow-derived), lacking the Epstein-Barr virus genome and derived from three human lymphomas. Proc Natl Acad Sci U S A 71: 3283–3286. 4369887 10.1073/pnas.71.8.3283 PMC388669

[56] Abe M, Nozawa Y, Wakasa H, Ohno H, Fukuhara S (1988) Characterization and comparison of two newly established Epstein-Barr virus-negative lymphoma B-cell lines. Surface markers, growth characteristics, cytogenetics, and transplantability. Cancer 61: 483–490. 3338018 10.1002/1097-0142(19880201)61:3<483::aid-cncr2820610313>3.0.co;2-l

[57] Tweeddale ME, Lim B, Jamal N, Robinson J, Zalcberg J, et al (1987) The presence of clonogenic cells in high-grade malignant lymphoma: a prognostic factor. Blood 69: 1307–1314. 3567358

[58] McCall EJ, Danielsson A, Hardern IM, Dartsch C, Hicks R, et al (2005) Improvements to the throughput of recombinant protein expression in the baculovirus/insect cell system. Protein Expr Purif 42: 29–36 S1046-5928(05)00109-9 [pii]; 10.1016/j.pep.2005.03.021 [doi] 15939290

[59] de Boer E, Rodriguez P, Bonte E, Krijgsveld J, Katsantoni E, et al (2003) Efficient biotinylation and single-step purification of tagged transcription factors in mammalian cells and transgenic mice. Proc Natl Acad Sci U S A 100: 7480–7485. 12802011 10.1073/pnas.1332608100 PMC164612

