# Supplementary figures and images for: MALT1 Auto-Proteolysis Is Essential for NF-κB-Dependent Gene Transcription in Activated Lymphocytes
Source: PLoS One. 2014 Aug 8;9(8):e103774. doi: 10.1371/journal.pone.0103774 (PMC4126661; doi:10.1371/journal.pone.0103774)

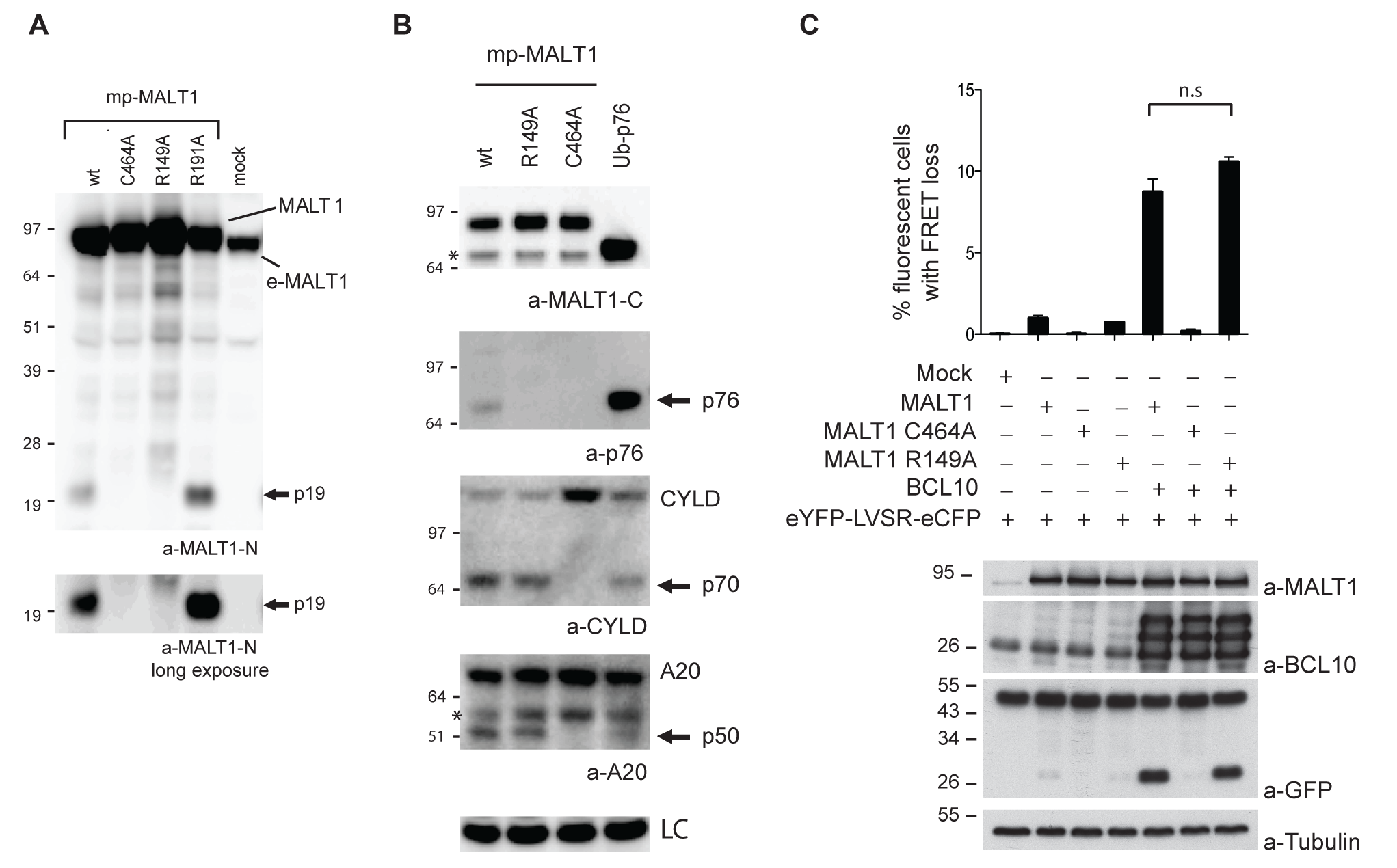

Supplement: Figure S1 — MALT1 cleavage does not affect its protease activity. A) Immunoblot of lysates of 293T cells transiently expressing mp-MALT1 and its mutants as specified with a-MALT1-N. eMALT1: endogenous MALT1. Arrow indicates the N-terminal p19 cleavage fragment. All molecular mass standards are in kDa. B) Immunoblot of lysates of 293T cells transiently expressing mp-MALT1, its R149A and C464A mutants, and Ubiquitin-p76 with indicated antibodies. Arrows indicate the MALT1 (p76), CYLD (p70) and A20 (p50) cleavage fragments respectively. * non-specific fragments. LC: a-specific fragment detected with the p76 neo-epitope antibody used as loading control. C) HEK293T cells were transfected with the eYFP–Leu-Val-Ser-Arg–eCFP probe (eYFP-LVSR-eCFP) and the indicated constructs. Probe cleavage (as gain in eCFP fluorescence, labeled as “% of fluorescent cells with FRET loss” in the y-axis of the graph) was assessed by flow cytometry, gating on eYFPhi cells (upper panel). In addition, cell lysates were analyzed by blotting for MALT1, BCL10, GFP and Tubulin, as indicated (lower panel). Compared to flow cytometry, in which only eYFPhi cells are included in the analysis, the Western blot analysis shows a higher percentage of reporter cleavage because all cells (including cells expressing low levels of the reporter) are lysed and analyzed. (TIF) [file pone.0103774.s001.tif]

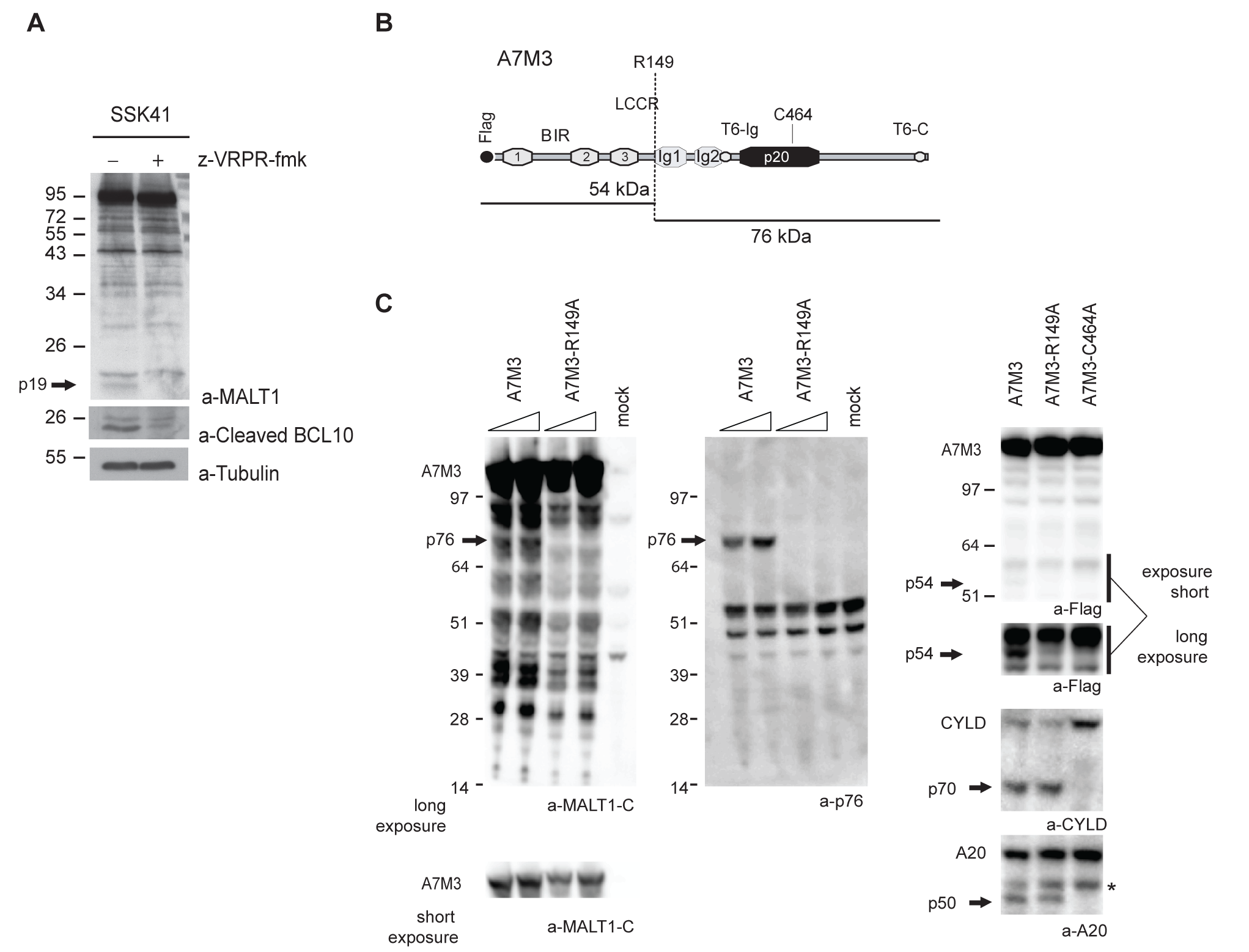

Supplement: Figure S2 — MALT1 and API2-MALT1 autoproteolysis in SSK41 lymphoma cells. A) Immunoblot of lysates from SSK41 cells, left untreated or treated with 50 µM z-VRPR-fmk (36h), with antibodies against MALT1, cleaved BCL10 and tubulin. Arrow indicates the MALT1 p19 cleavage fragment. B) Features of the A7M3 fusion variant of API2-MALT1 plus the domain content (solid bars) of the 53 and 76 kDa cleavage fragments. BIR: Baculovirus “inhibition of apoptosis” repeat. C) Immunoblot analysis of lysates of 293T cells transiently expressing increasing concentrations of Flag-tagged A7M3 and A7M3-R149A mutant, indicating the p76 C-terminal fragment detected with a-MALT1-C (left) or the p76 neo-epitope specific antibody (middle). Right: Immunoblot analysis of 293T cells transiently expressing the API2-MALT1 fusion variant A7M3, A7M3-R149A and A7M3-C464A with antibodies against CYLD and A20. Arrows indicate their respective p70 and p50 cleavage fragments. Immunoblot with the Flag antibody (N-terminus) was performed to demonstrate equal expression of A7M3 or its mutants and shows the N-terminal cleavage fragment (p54) of A7M3. * non-specific fragment. (TIF) [file pone.0103774.s002.tif]

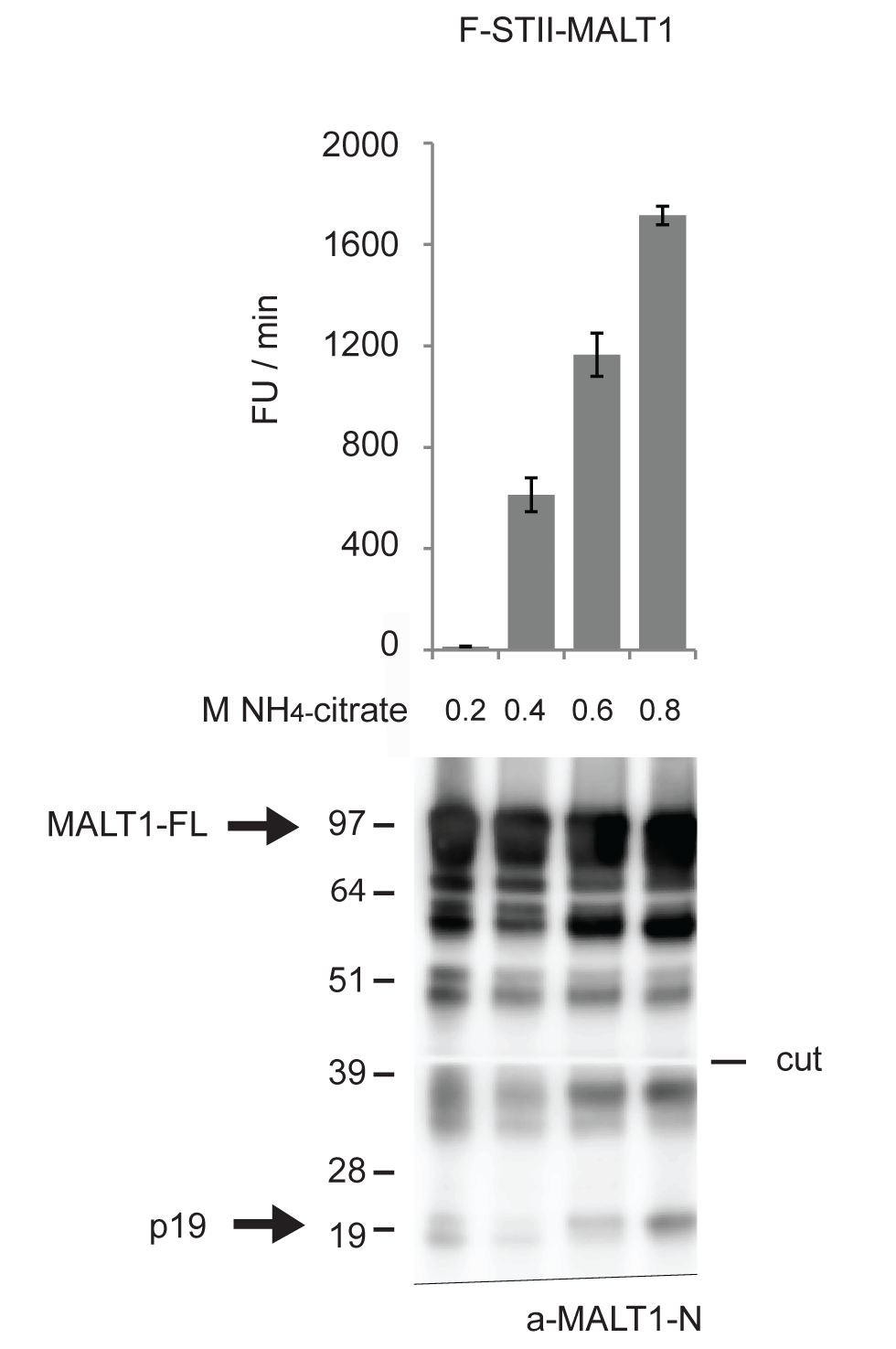

Supplement: Figure S3 — MALT1 undergoes auto-proteolysis in vitro . Top: In vitro cleavage of the fluorogenic tetrapeptide substrate Ac-LVSR-AMC (50 µM) by F-STII-MALT1 in increasing concentrations of the cosmotropic salt NH4-citrate (0.2, 0.4, 0.6, and 0.8 M). The barchart shows cleavage activity as Fluorescence Units (FU) increase/min. Results are expressed as means ± SD (n = 3). Bottom: enzymatic reactions were analysed by immunoblotting with a-MALT1-N. The blot was previously cut in two to detect p76 and p19 separately, which explains the white line in the middle. (TIF) [file pone.0103774.s003.tif]

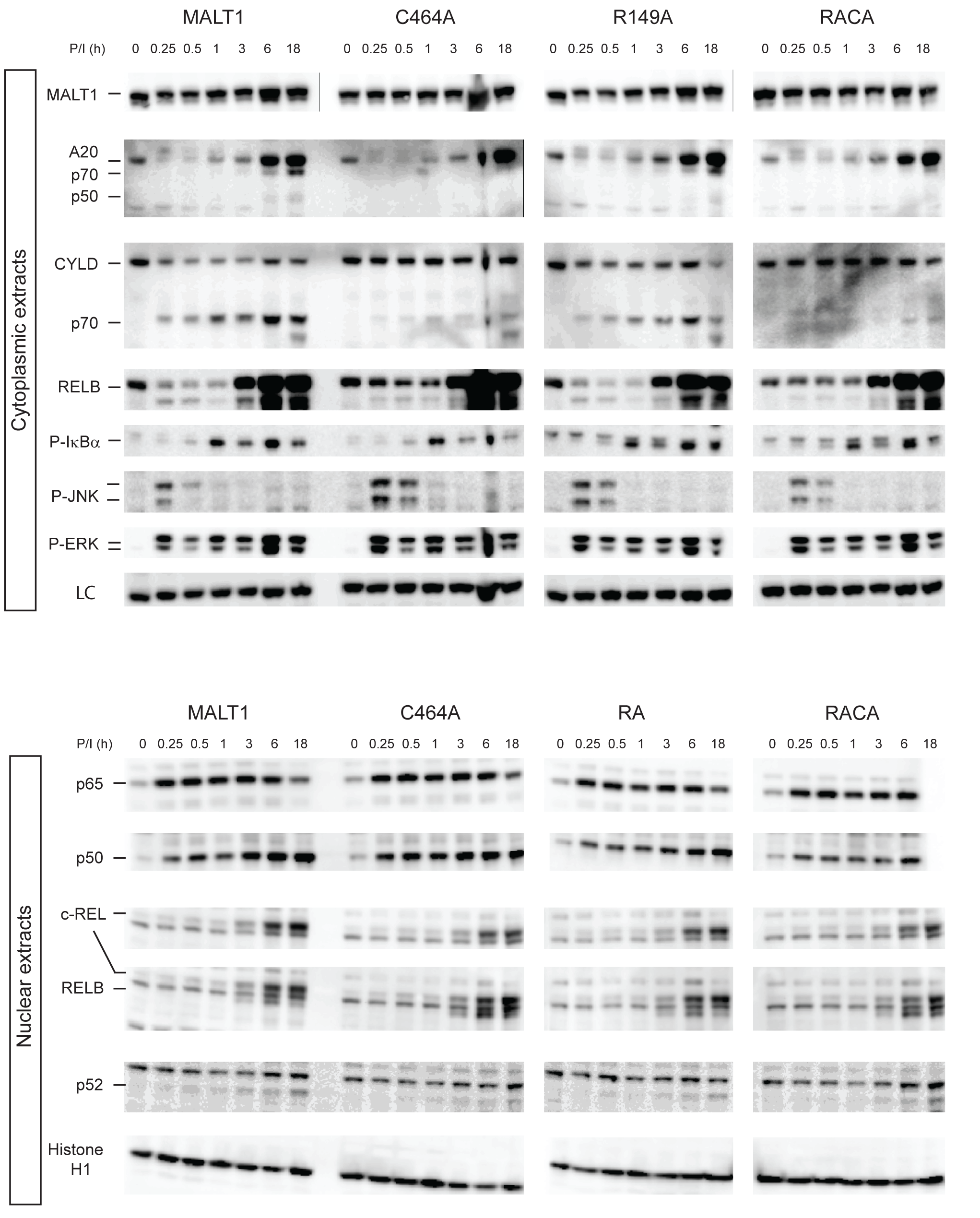

Supplement: Figure S4 — MALT1 auto-proteolysis is not required for initial IκBα phosphorylation and NF-κB nuclear translocation in Jurkat T cells overexpressing MALT1 mutants. Jurkat T cells expressing MALT1 or the mutants C464A, R149A and RACA were stimulated with P/I for indicated times and cytosolic and nuclear extracts were immunoblotted with indicated antibodies. Blots used to detect c-Rel were re-used without stripping to detect RELB and therefore both bands are visible in the RELB panel (upper band = c-Rel, lower band = RELB). (TIF) [file pone.0103774.s004.tif]

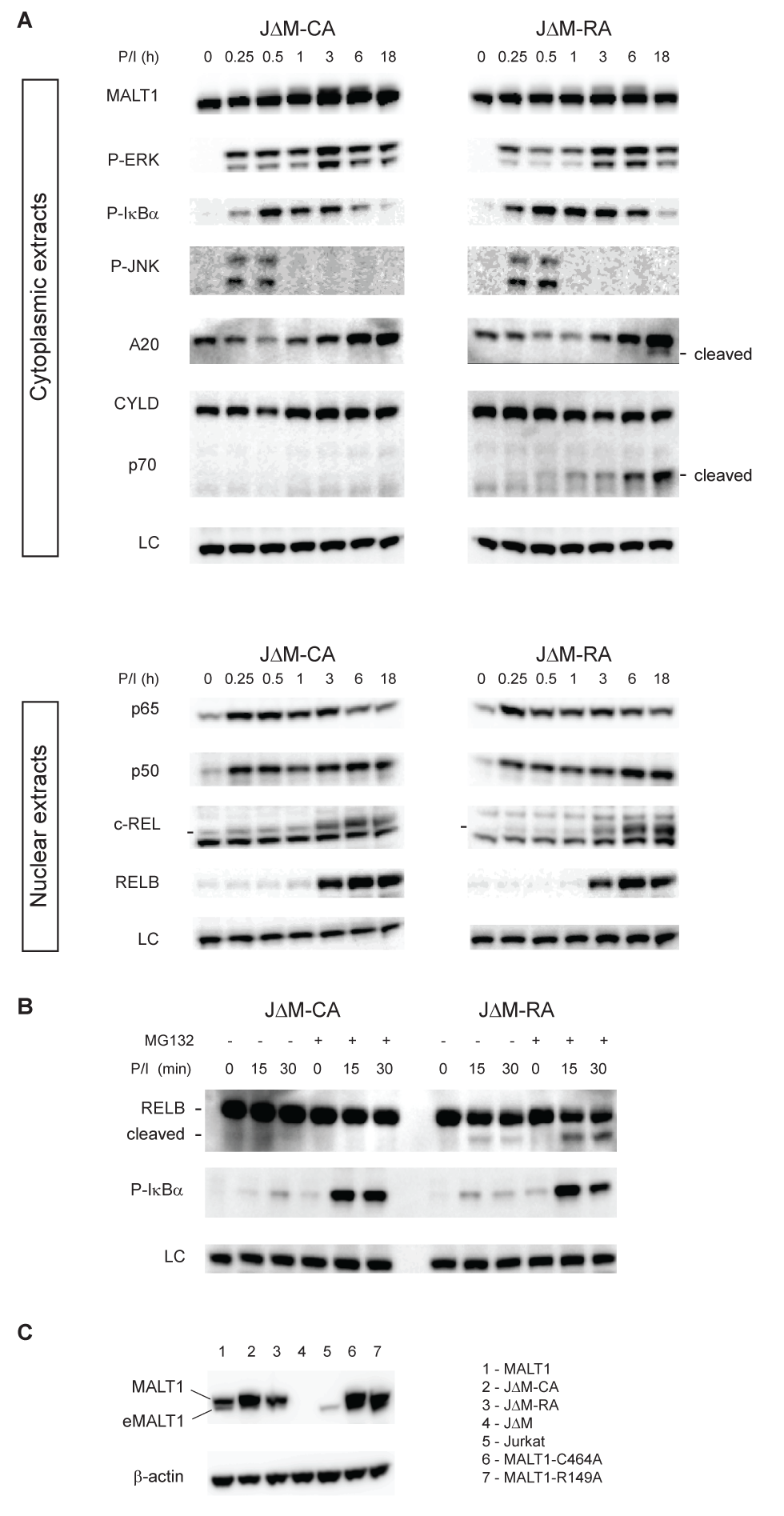

Supplement: Figure S5 — MALT1 auto-proteolysis is not required for initial IκBα phosphorylation and NF-κB nuclear translocation in JΔM-CA and JΔM-RA cells. A) Jurkat T cells expressing MALT1-C464A or MALT1-R149A were genetically modified with TALENs to inactivate endogenous MALT1 expression generating JΔM-CA and JΔM-RA cells respectively. Cells were stimulated with P/I for indicated times and cytosolic and nuclear extracts were immunoblotted with indicated antibodies. LC: a-specific band used as loading control. B) JΔM-CA and JΔM-RA cells were pre-treated with MG-132 for 30 min before stimulation for 15 or 30 min with PMA/ionomycin (P/I). Total cell lysates were immunoblotted with indicated antibodies. LC: a-specific band used as loading control. C) Immunoblot with a-MALT1-N showing expression of ectopic MALT1 and mutants relative to endogenous MALT1 (lane 5) in the different Jurkat cell lines. β-actin: loading control. (TIF) [file pone.0103774.s005.tif]

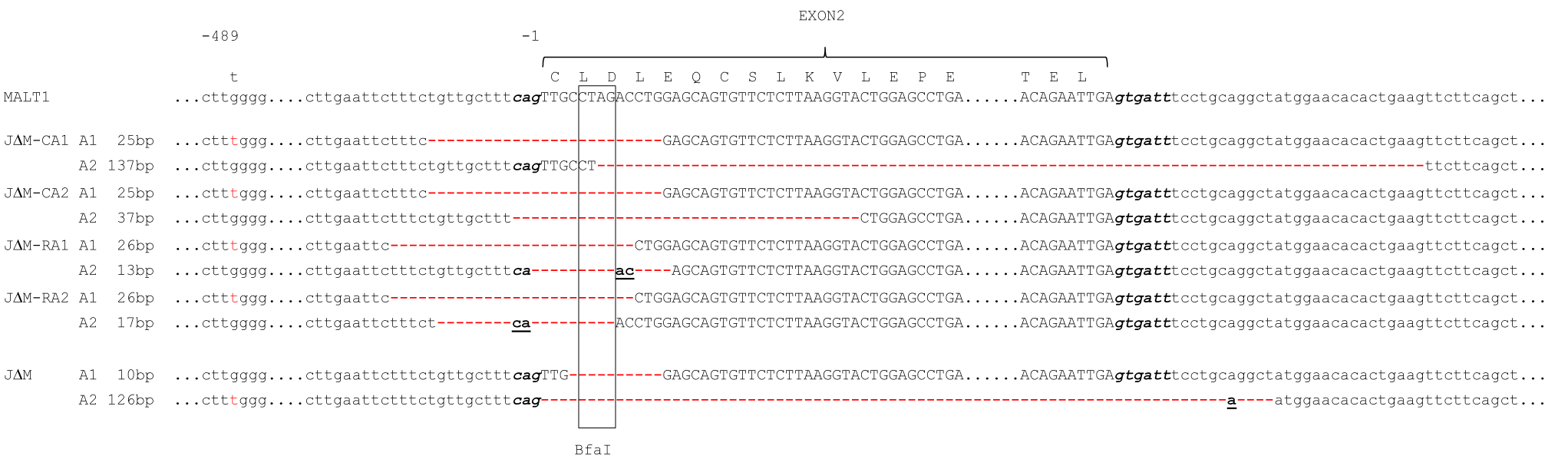

Supplement: Figure S6 — TALEN-mediated knock-out of endogenous MALT1. Jurkat T cells and Jurkat T cells with ectopic expression of MALT1-R149A and MALT1-C464A were electroporated with TALEN pairs targeting a BfaI at the intron1-exon2 boundary of MALT1. Position and size of the introduced deletions in the different generated cell lines are indicated. A single nucleotide polymorphism located 489 bp upstream of exon 2 of MALT1 was used to discriminate the 2 MALT1 alleles. (TIF) [file pone.0103774.s006.tif]
